# Supplementary material for: Ethanol Extract of Adlay Hulls Suppresses Acute Myeloid Leukemia Cell Proliferation via PI3K/Akt Pathway Inhibition
Source: Curr Issues Mol Biol. 2025 May 13;47(5):358. doi: 10.3390/cimb47050358 (PMC12109684; doi:10.3390/cimb47050358)
Supplement: Supplementary file 1 [file cimb-47-00358-s001.zip › Supplement Material.pdf]

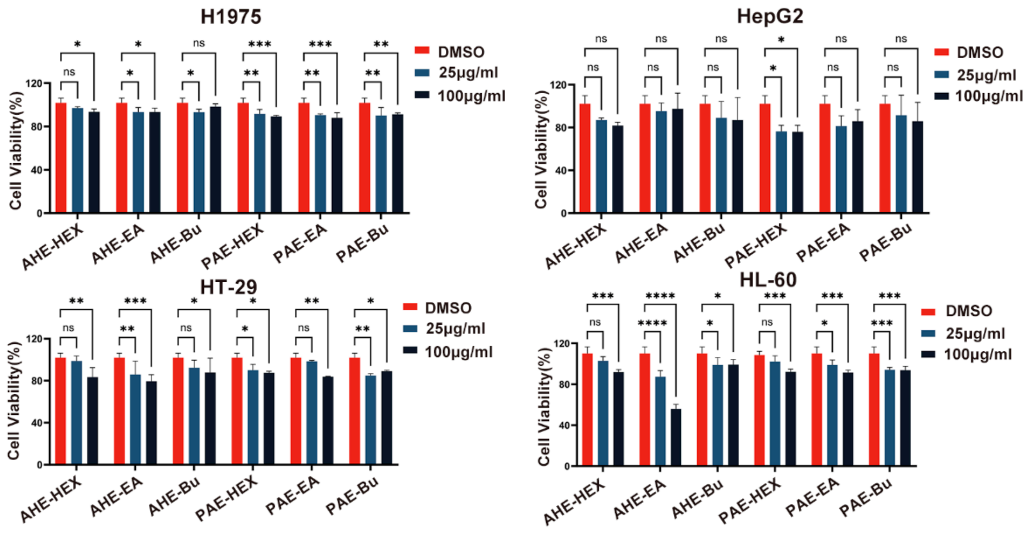

Figure S1. Inhibitory effects of AHE and PAE subfractions on cell viability in H1975, HepG2, HT29 and HL-60 cell lines.

Cell viability of H1975, HepG2, HT29 and HL-60 cells treated with different subfractions of AHE-EA (AHE-HEX, AHE-EA and AHE-Bu) and PAE (PAE-HEX, PAE-EA, and PAE-Bu) at concentrations of 25 μg/ml and 100 μg/ml was measured using the CCK-8 assay after a 72-hour treatment. DMSO was used as the control. All data are shown as mean±SD, n = 3, two-way ANOVA, *p < 0.05, **p < 0.01, ***p < 0.001, ****p < 0.0001, ns=not significant.

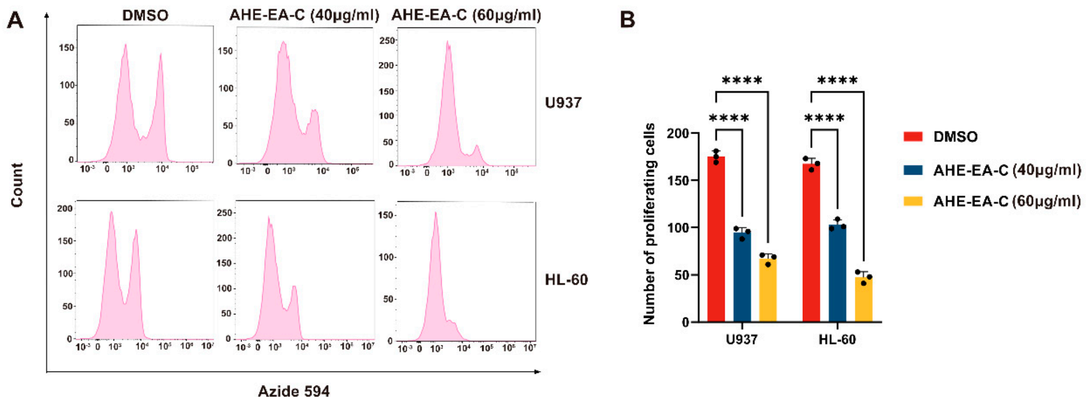

Figure S2. AHE-EA-C inhibits DNA synthesis in AML cells as assessed by EdU incorporation assay. (A) U937 and HL-60 cells were treated with DMSO or increasing concentrations of AHE-EA-C (40 and 60 μg/mL) for 48 h, followed by incubation with

10 μM EdU for 2.5 h. Cells were subsequently stained with the BeyoClick™ EdU-594 Cell Proliferation Kit and analyzed by flow cytometry. (B) Statistical plot of the proportion of Edu-positive cells. All data are shown as mean±SD, $n = 3$, two-way ANOVA, ****$p < 0.0001$.

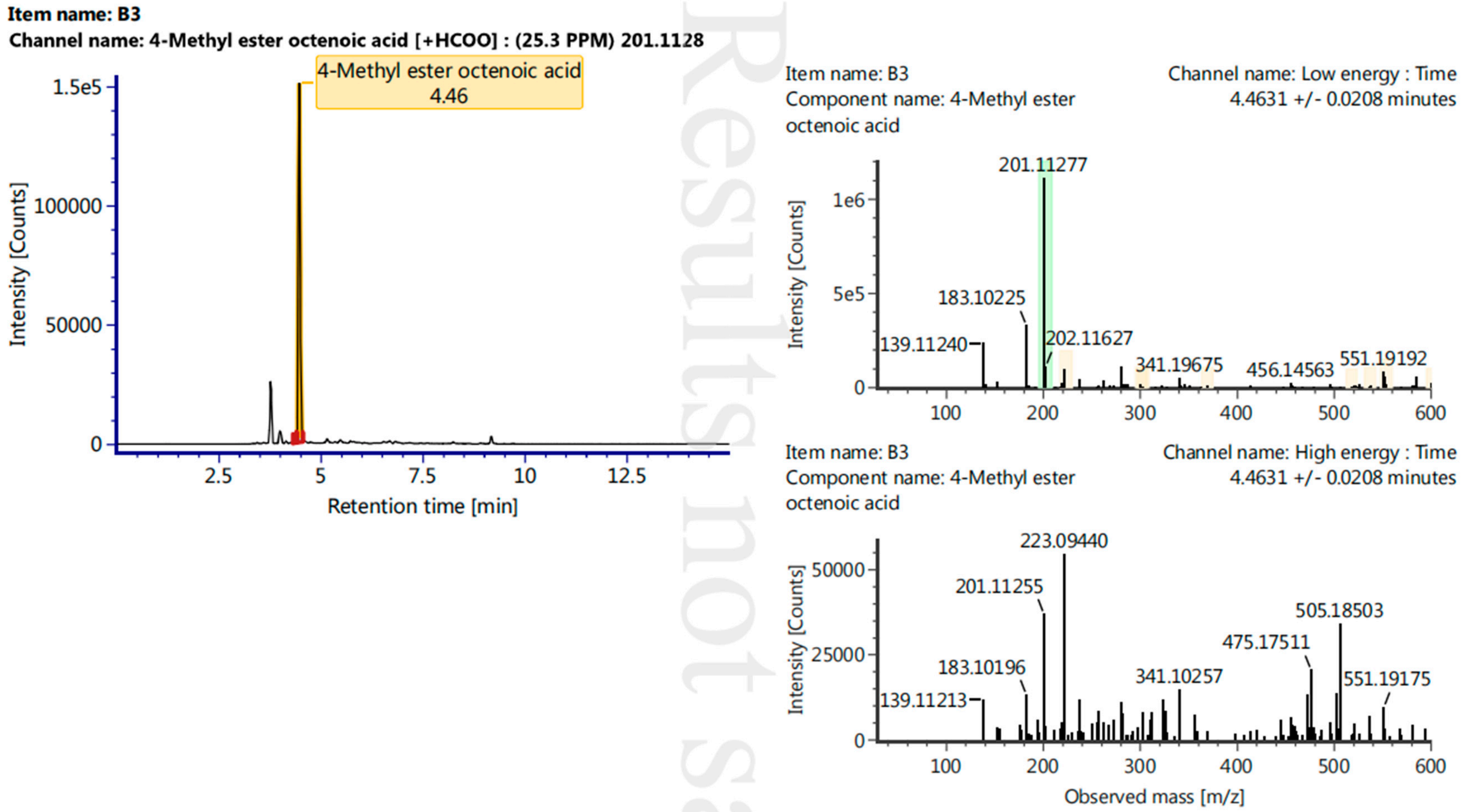

Figure S3. MS/MS spectrum of 4-Methyl ester octanoic acid

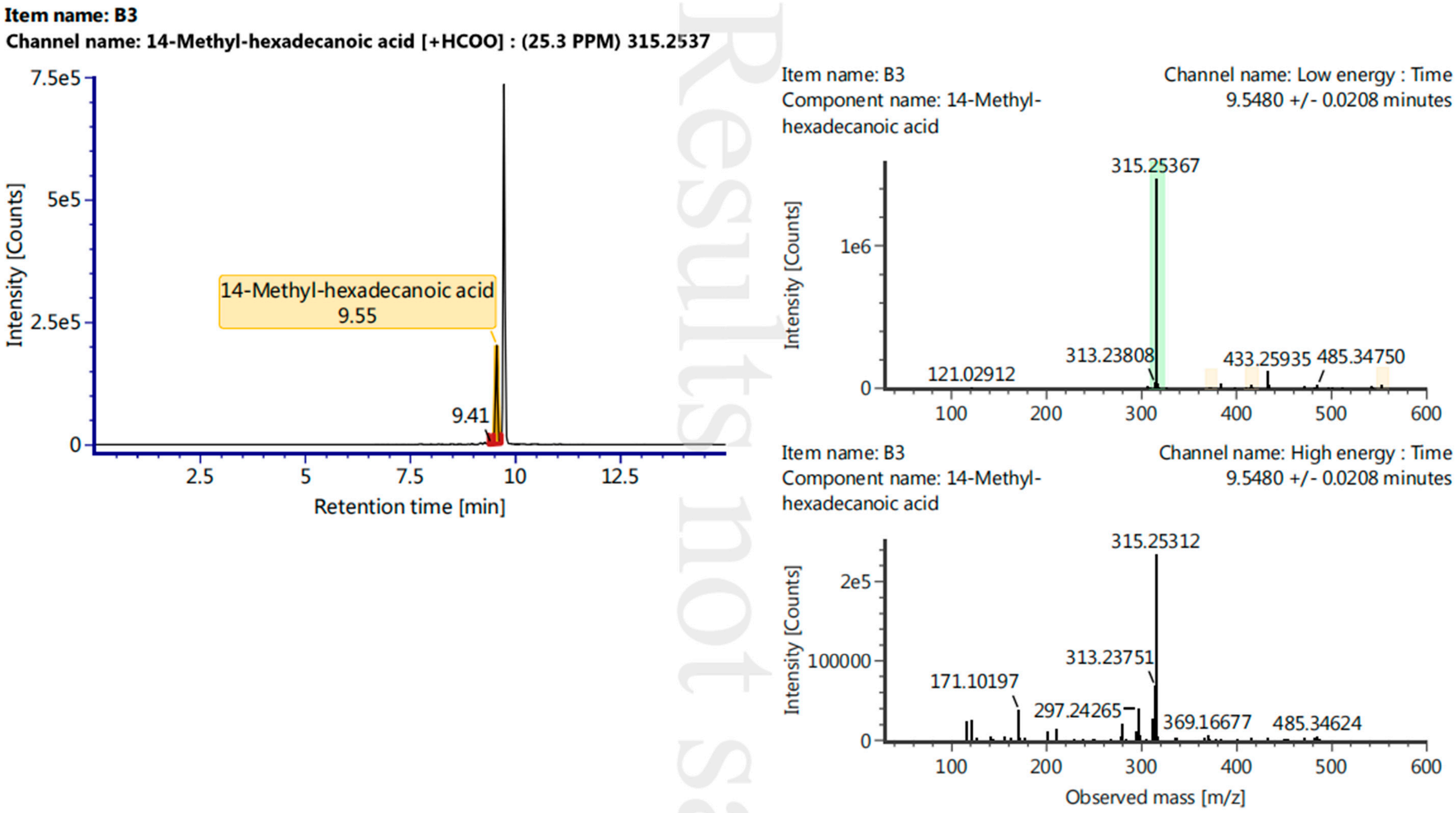

Results not Sa

Figure S4. MS/MS spectrum of 14-Methyl hexadecanoic acid

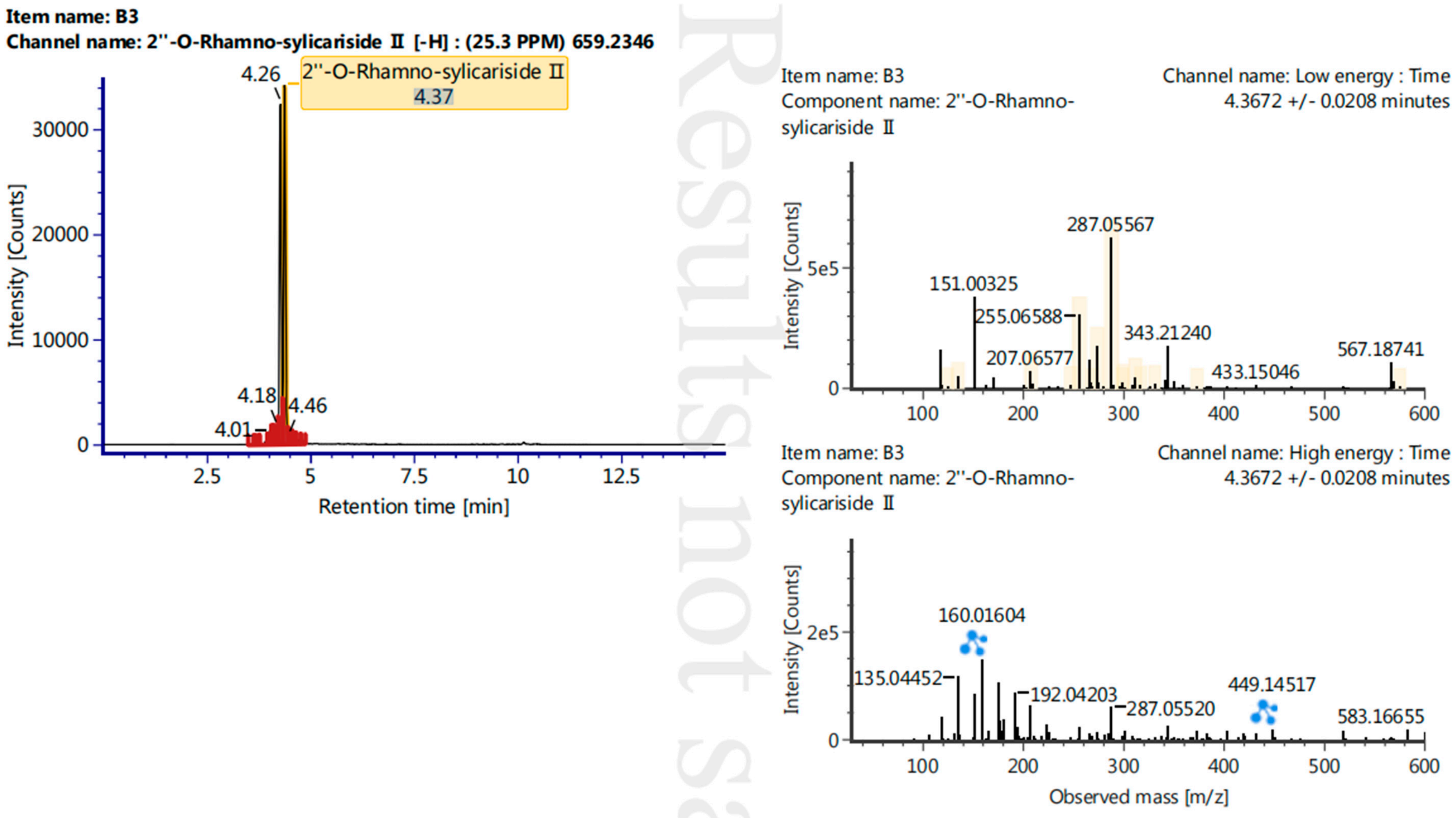

Figure S5. MS/MS spectrum of 2"-O-Rhamno sylicariside II

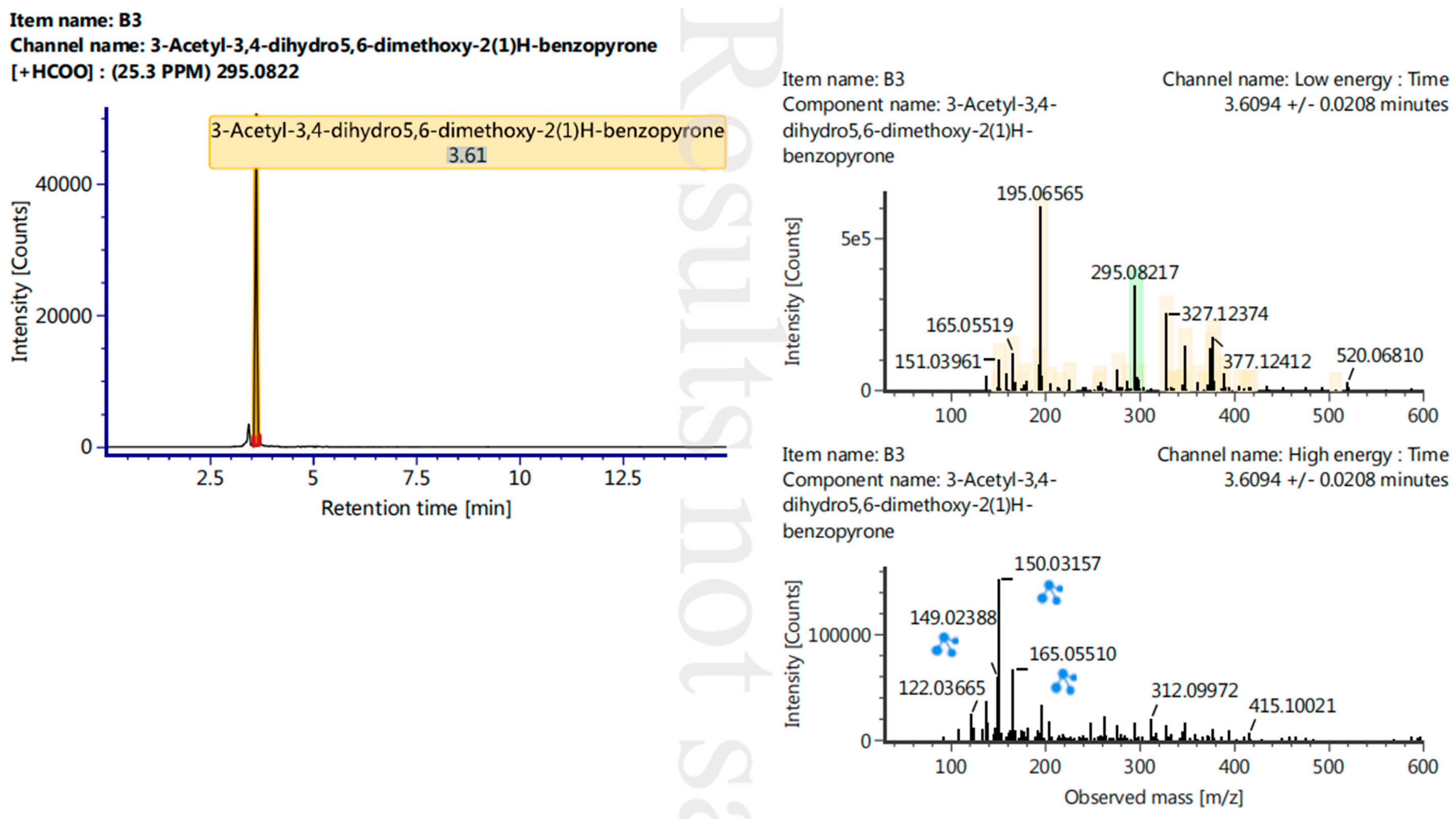

Figure S6. MS/MS spectrum of 3-Acetyl-3,4-dihydro5,6- dimethoxy-2(1)H-benzopyrone

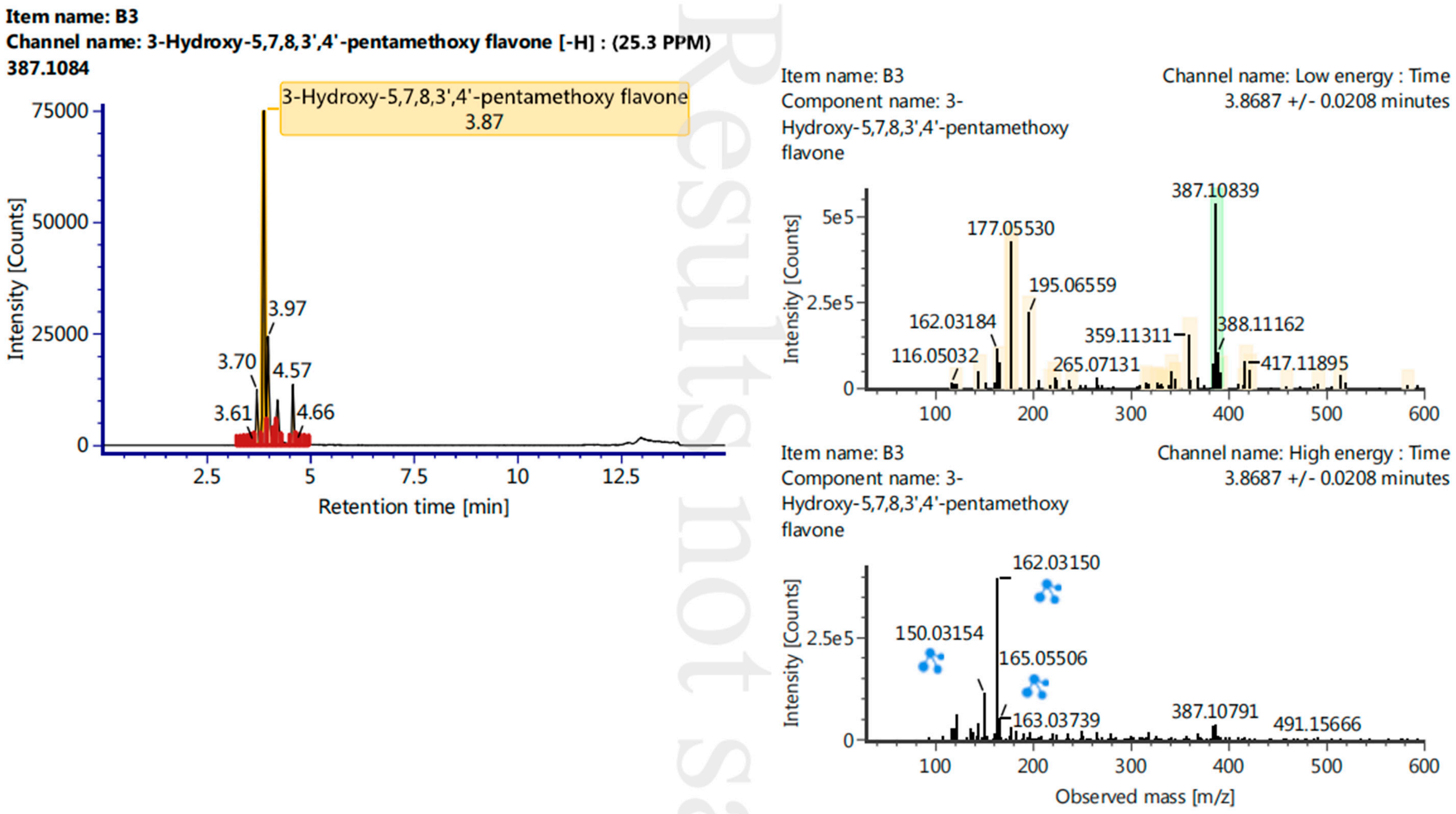

Results not Sa

Figure S7. MS/MS spectrum of 3-Hydroxy-5,7,8,3',4'-pentamethoxy flavone

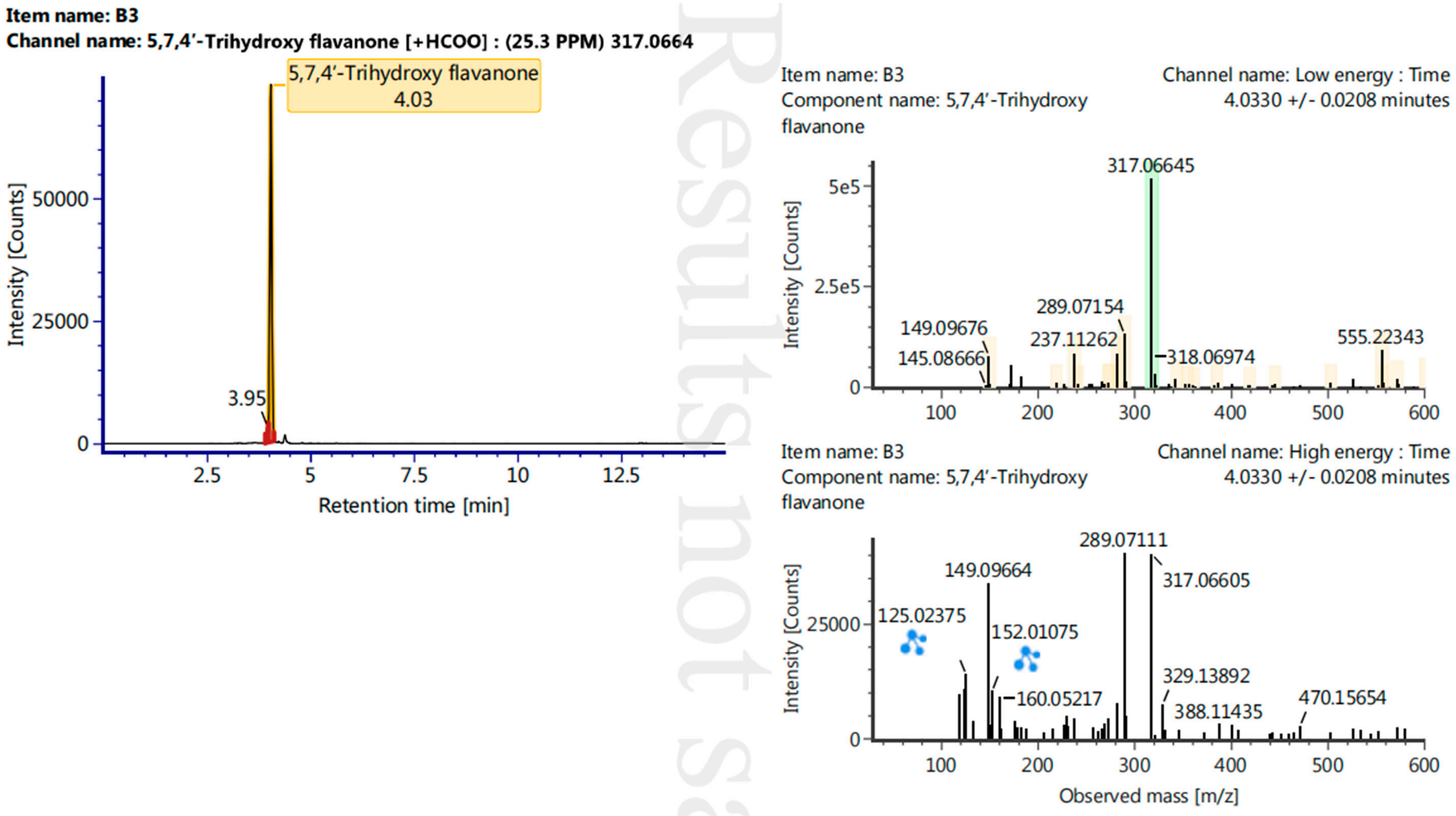

Figure S8. MS/MS spectrum of 5,7,4′-Trihydroxy flavanone

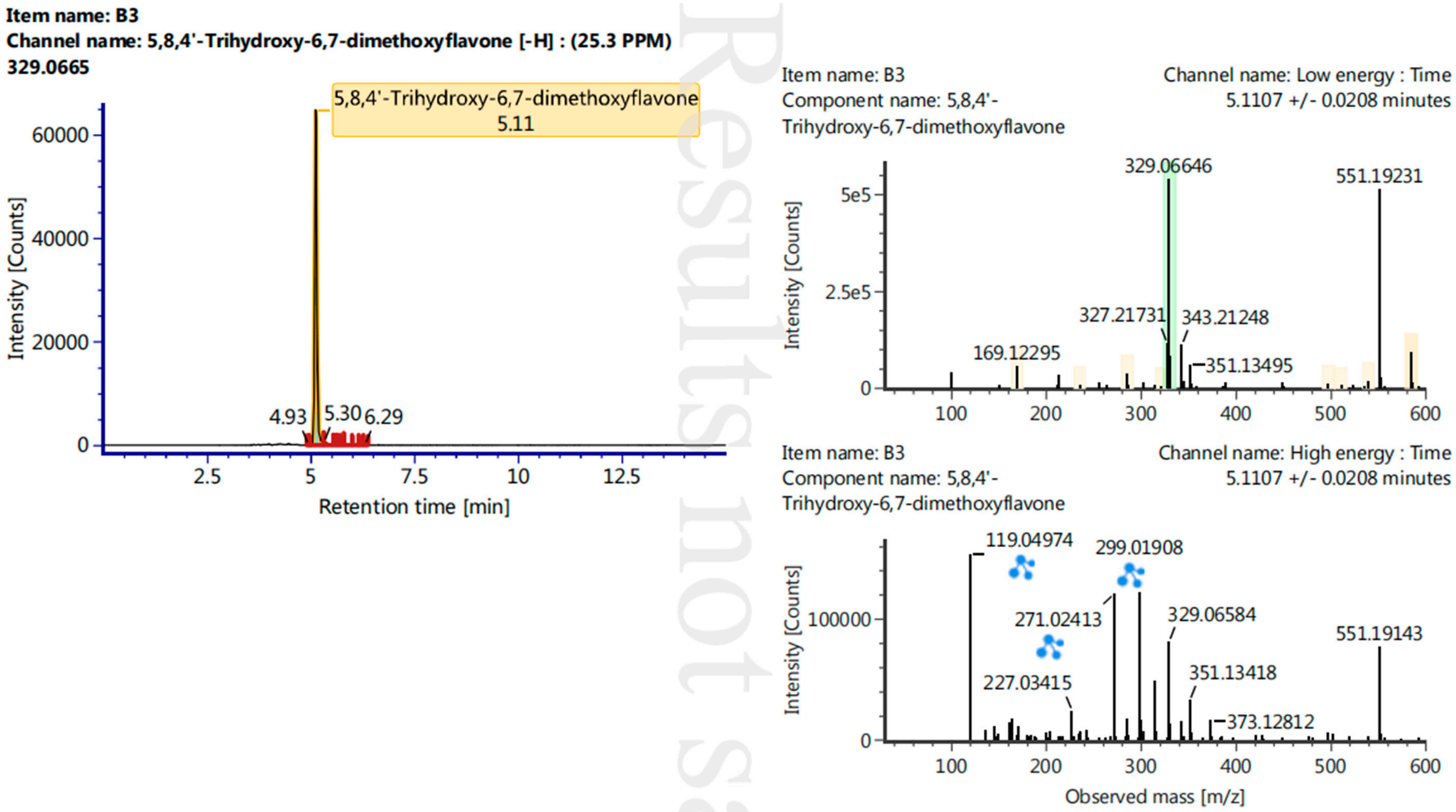

Results not Sa

Figure S9. MS/MS spectrum of 5,8,4'-Trihydroxy-6,7-dimethoxyflavone

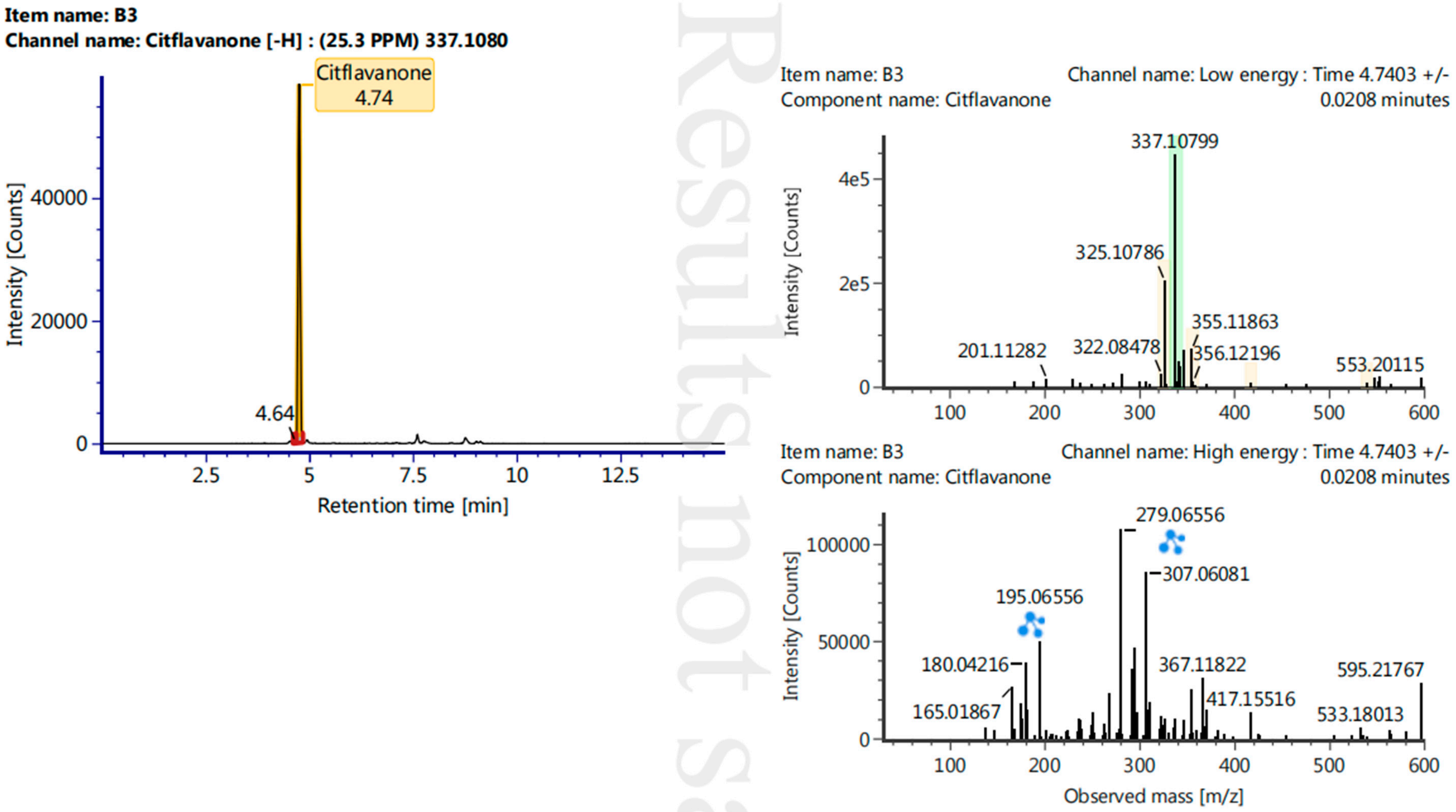

Figure S10. MS/MS spectrum of Citflavanone

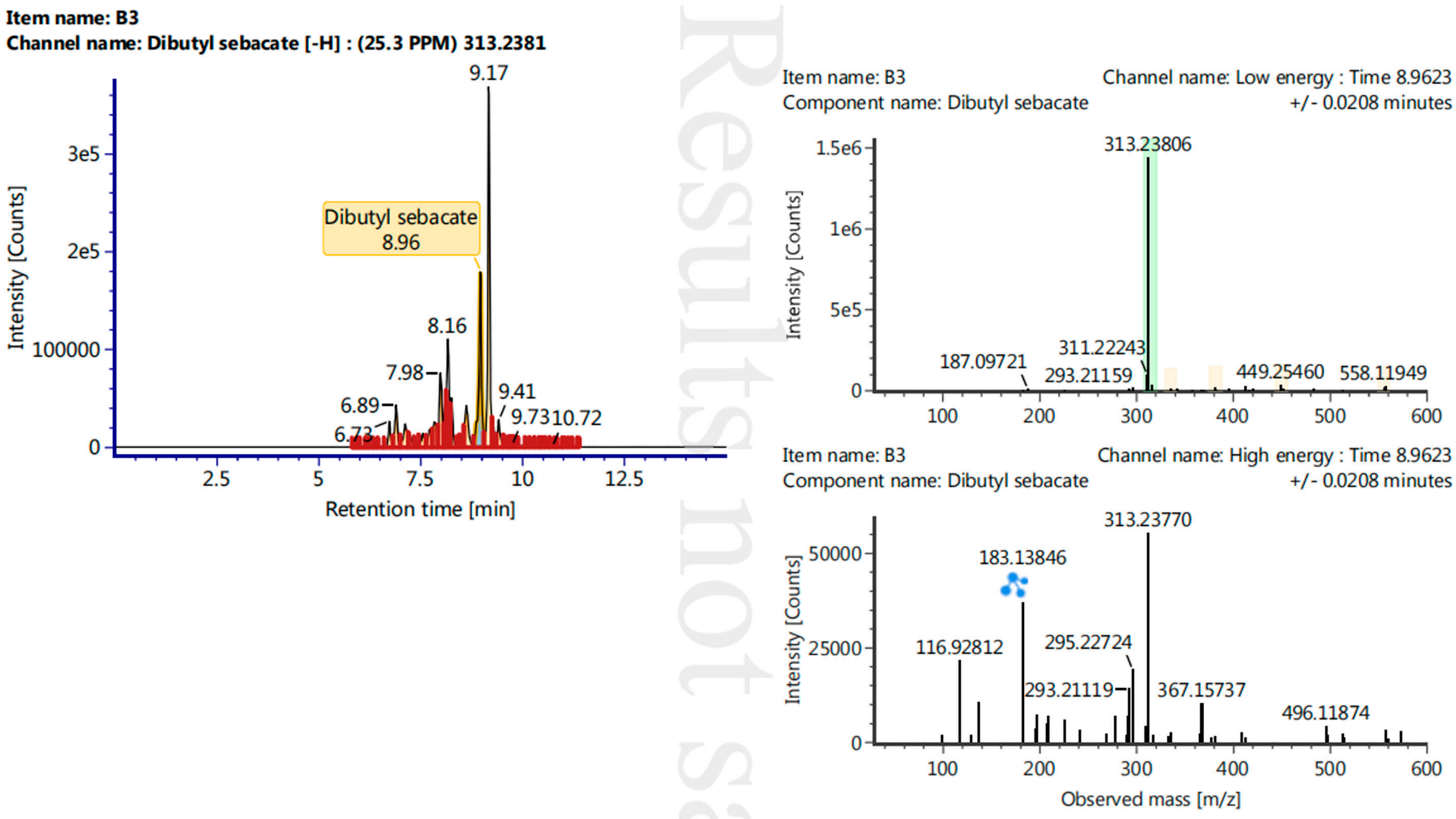

Figure S11. MS/MS spectrum of Dibutyl sebacate

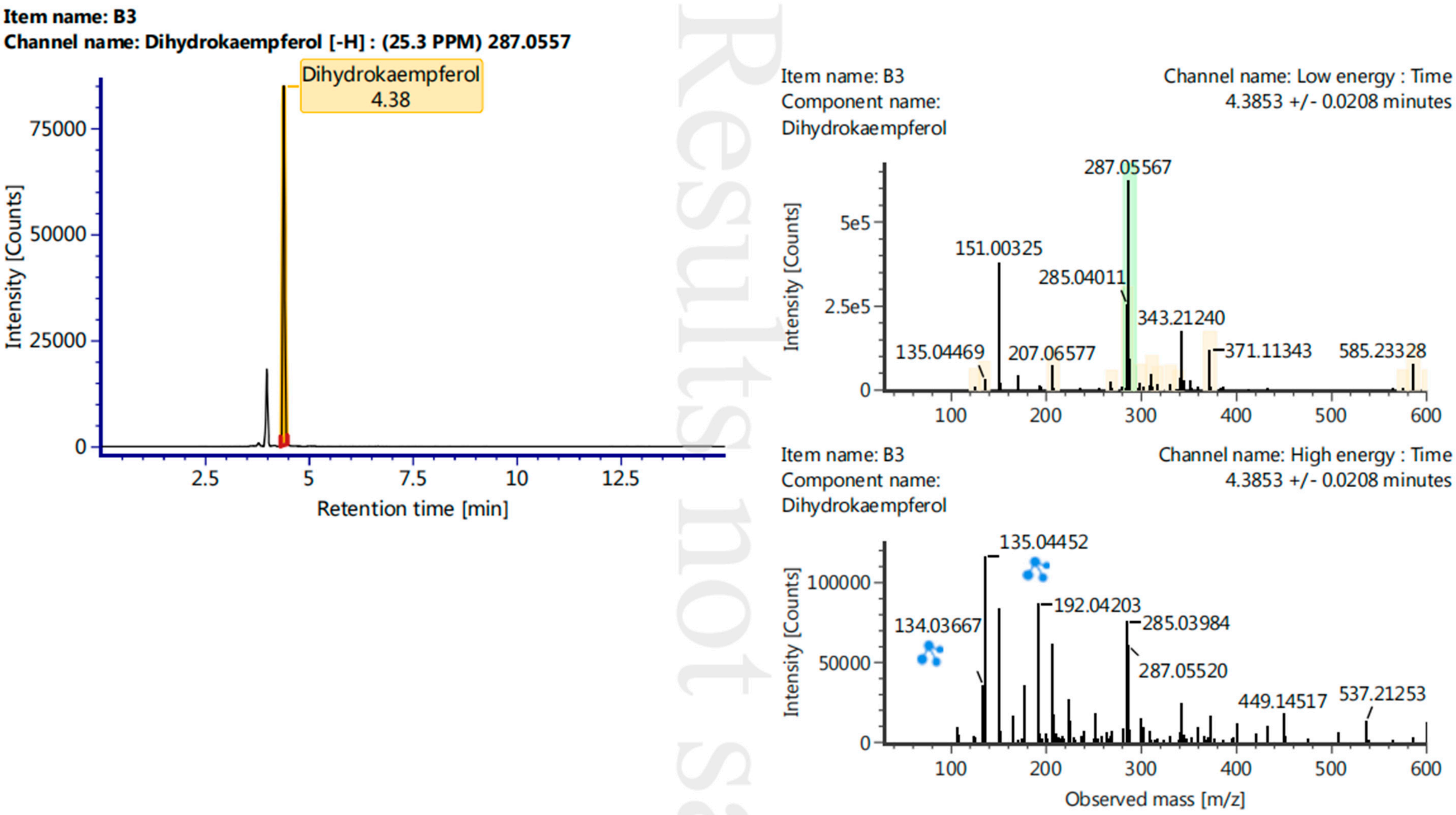

Results not Sa

Figure S12. MS/MS spectrum of Dihydrokaempferol

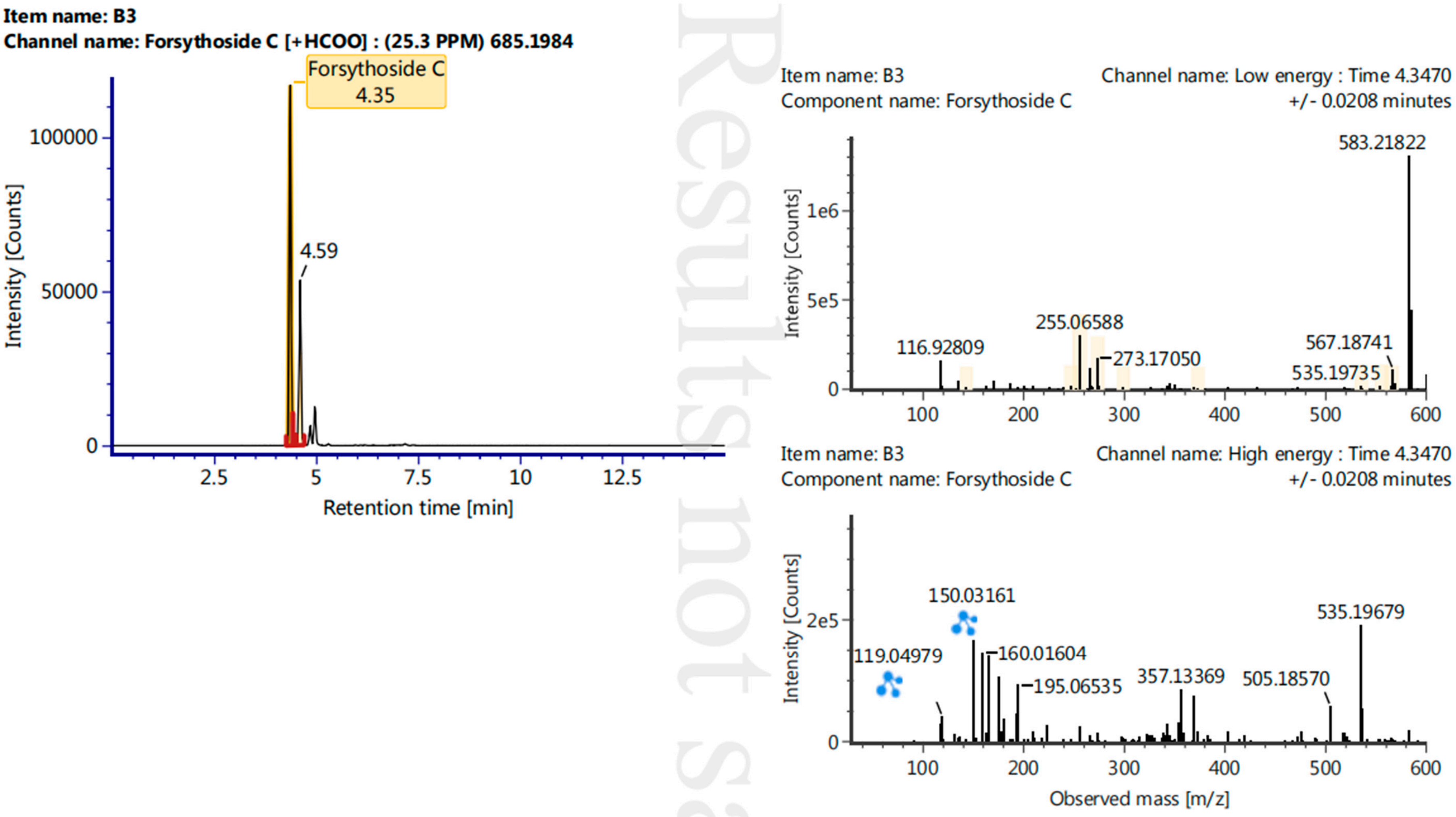

Figure S13. MS/MS spectrum of Forsythoside C

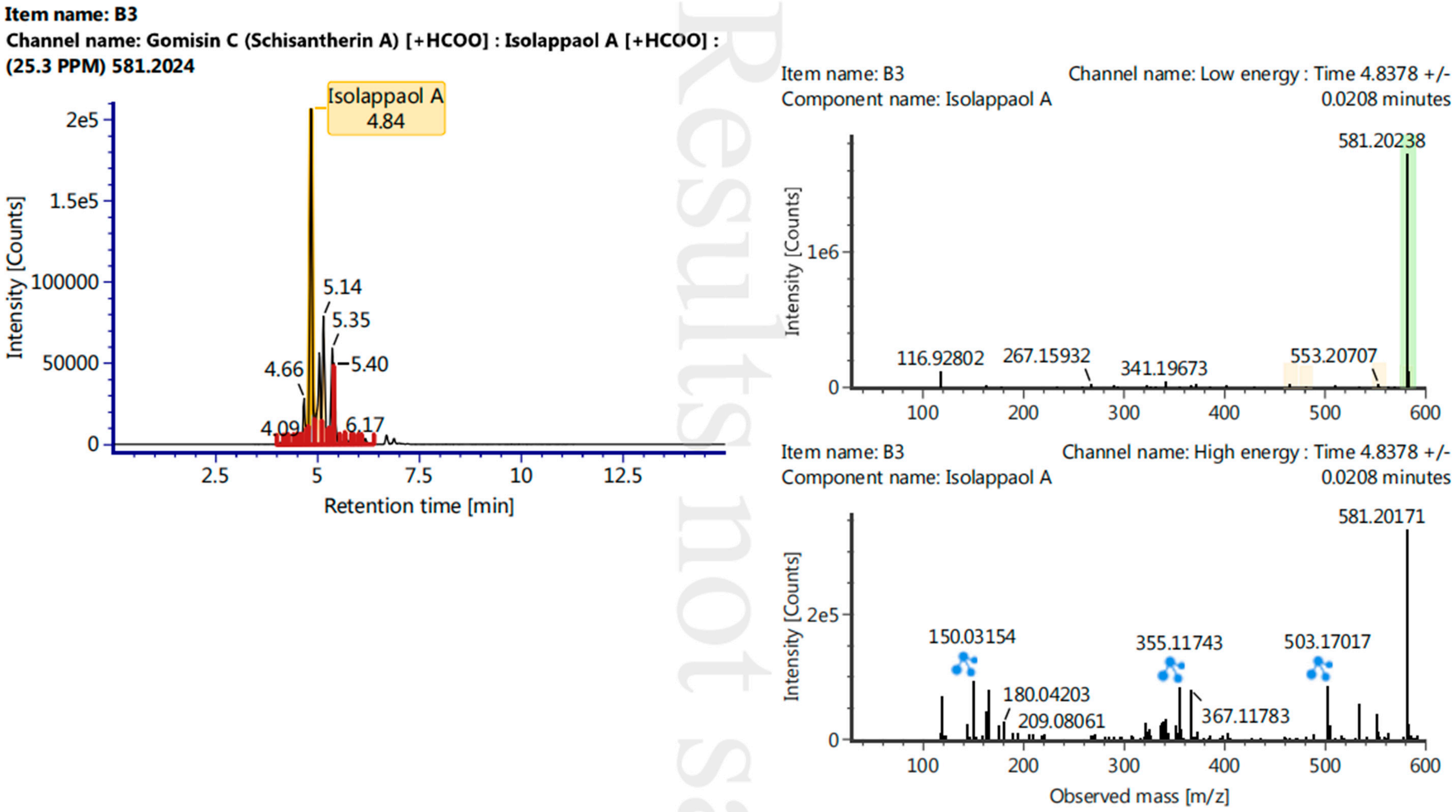

Results not Sa

Figure S14. MS/MS spectrum of Isolappaol A

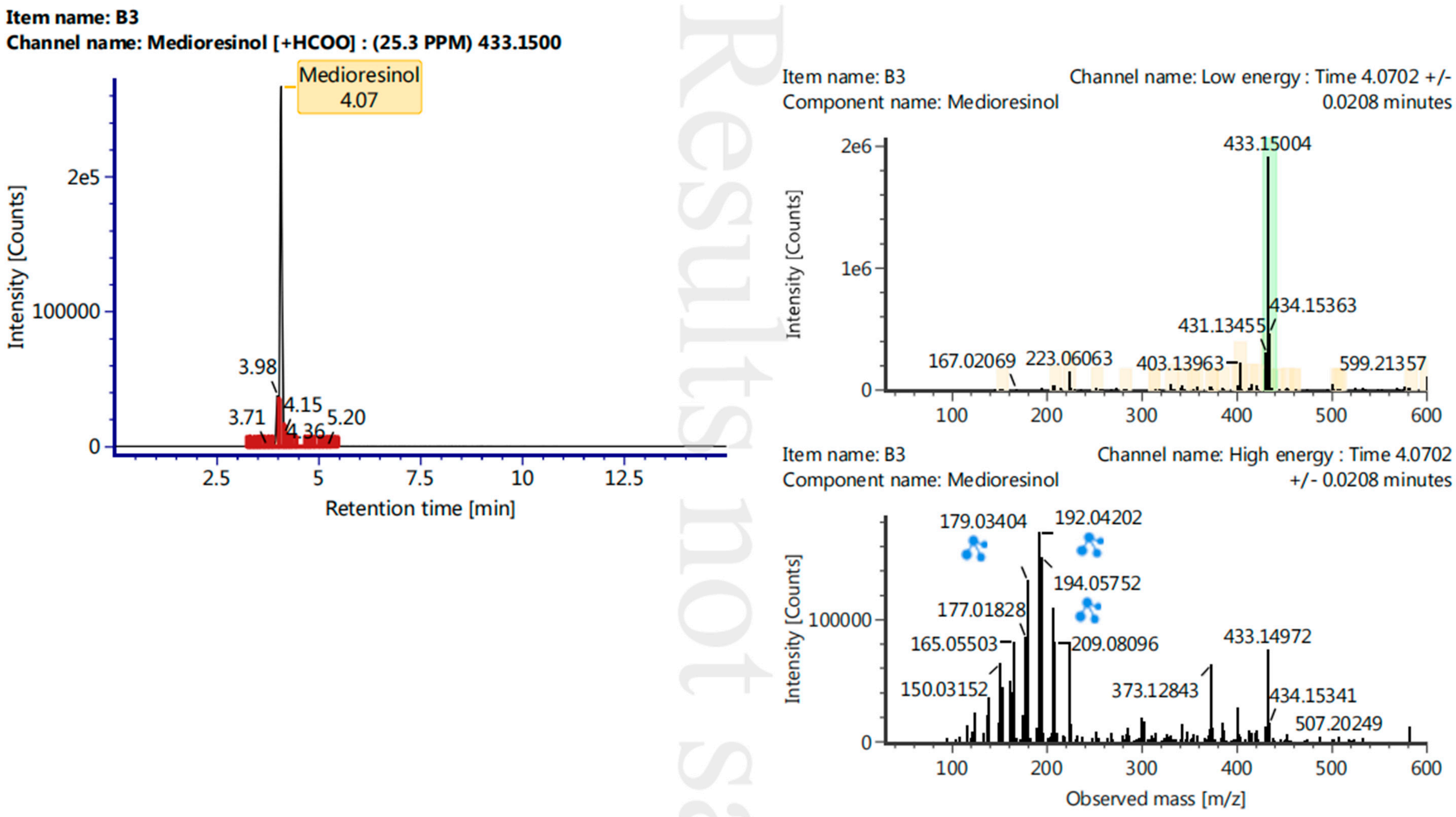

Figure S15. MS/MS spectrum of Medioresinol

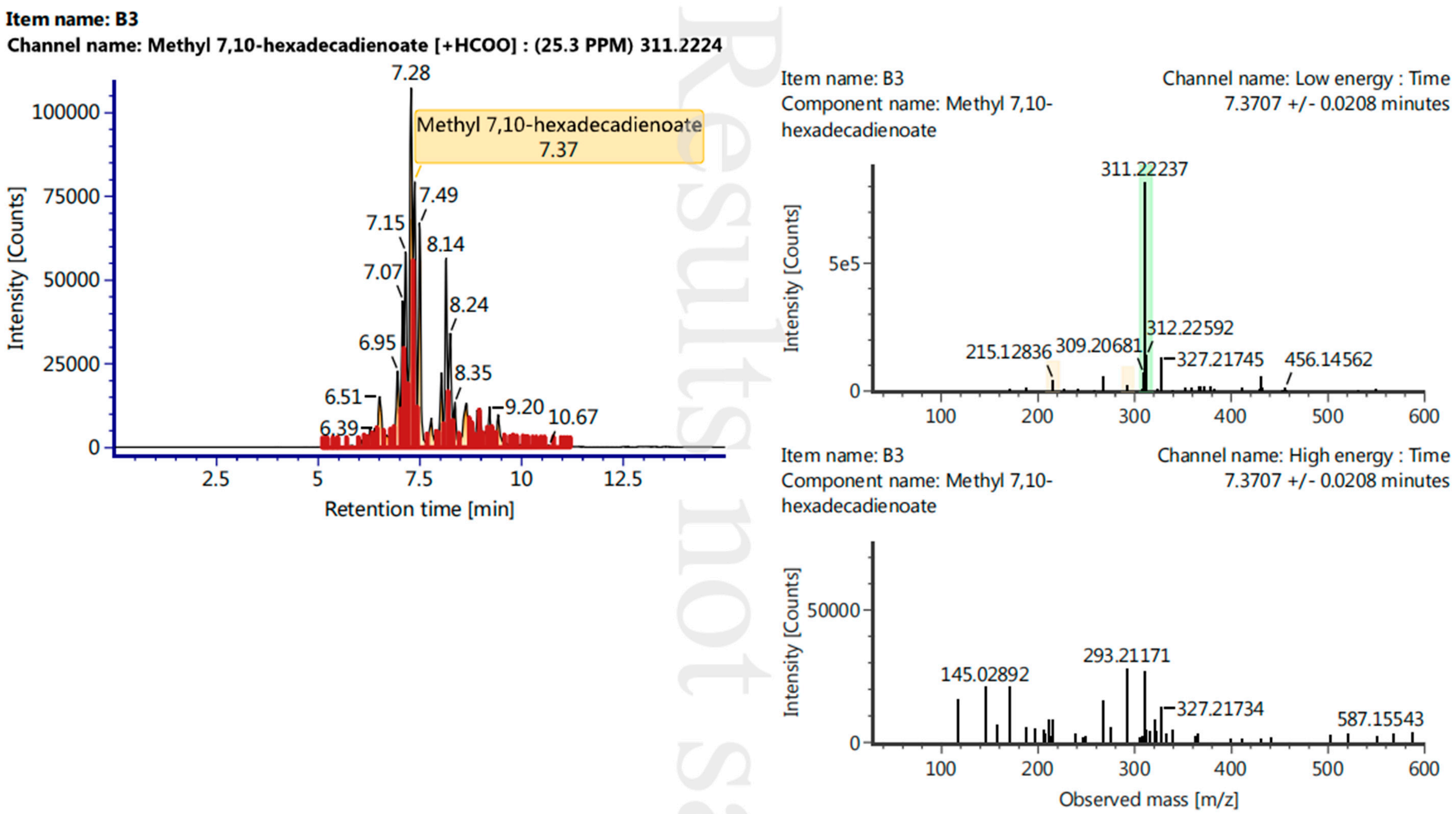

Figure S16. MS/MS spectrum of Methyl 7,10- hexadecadienoate

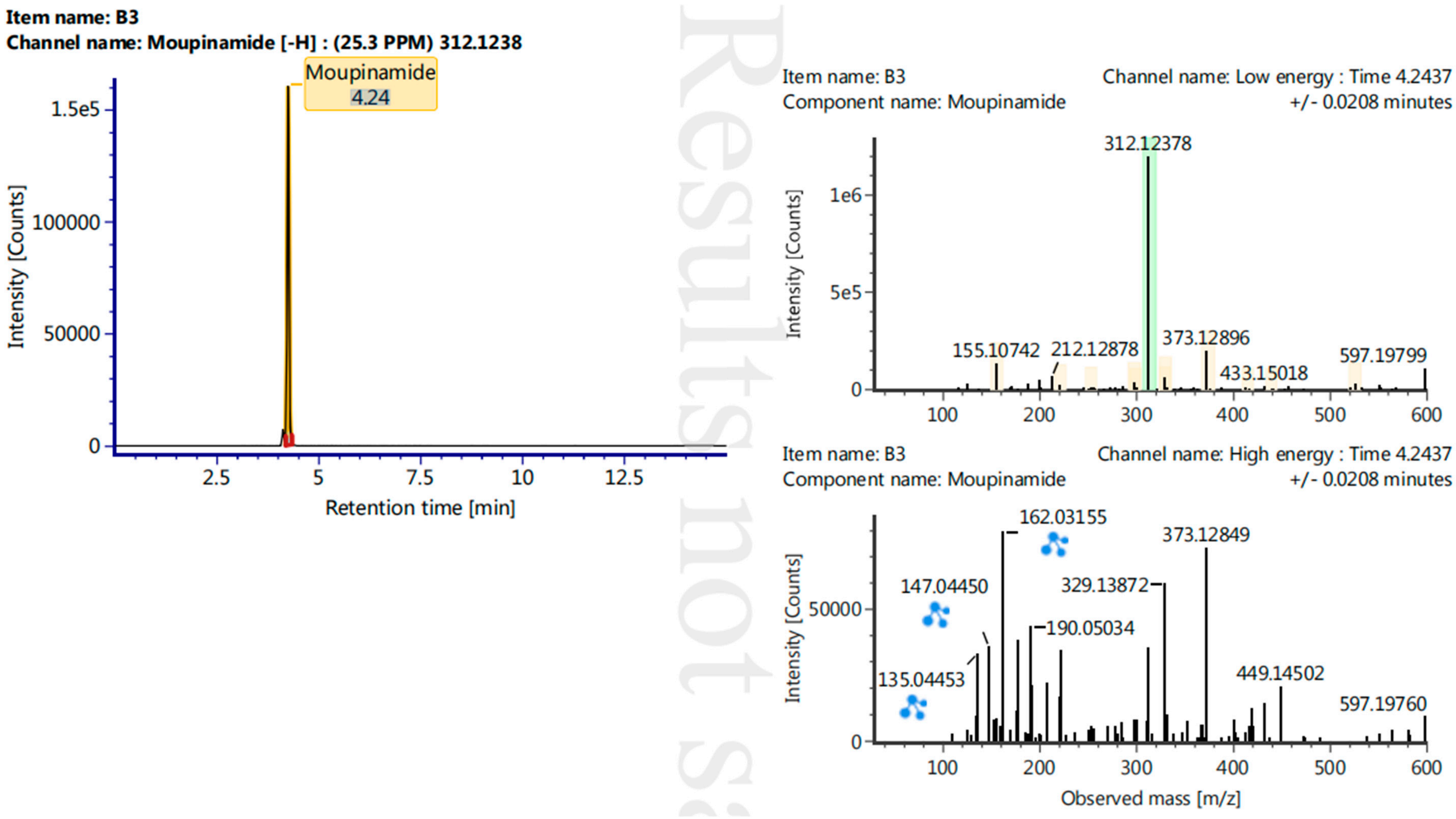

Figure S17. MS/MS spectrum of Moupinamide

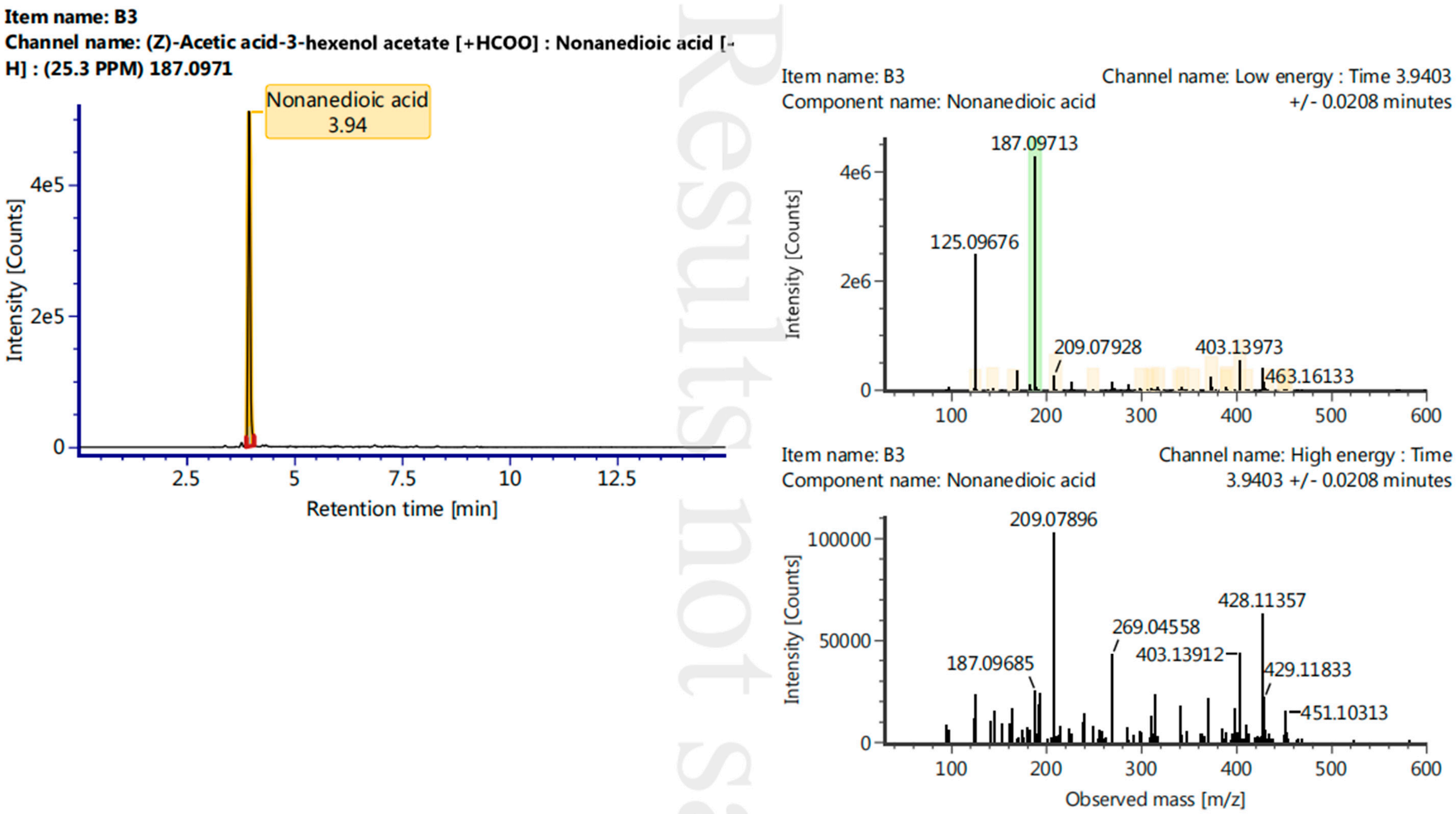

Figure S18. MS/MS spectrum of Nonanedioic acid

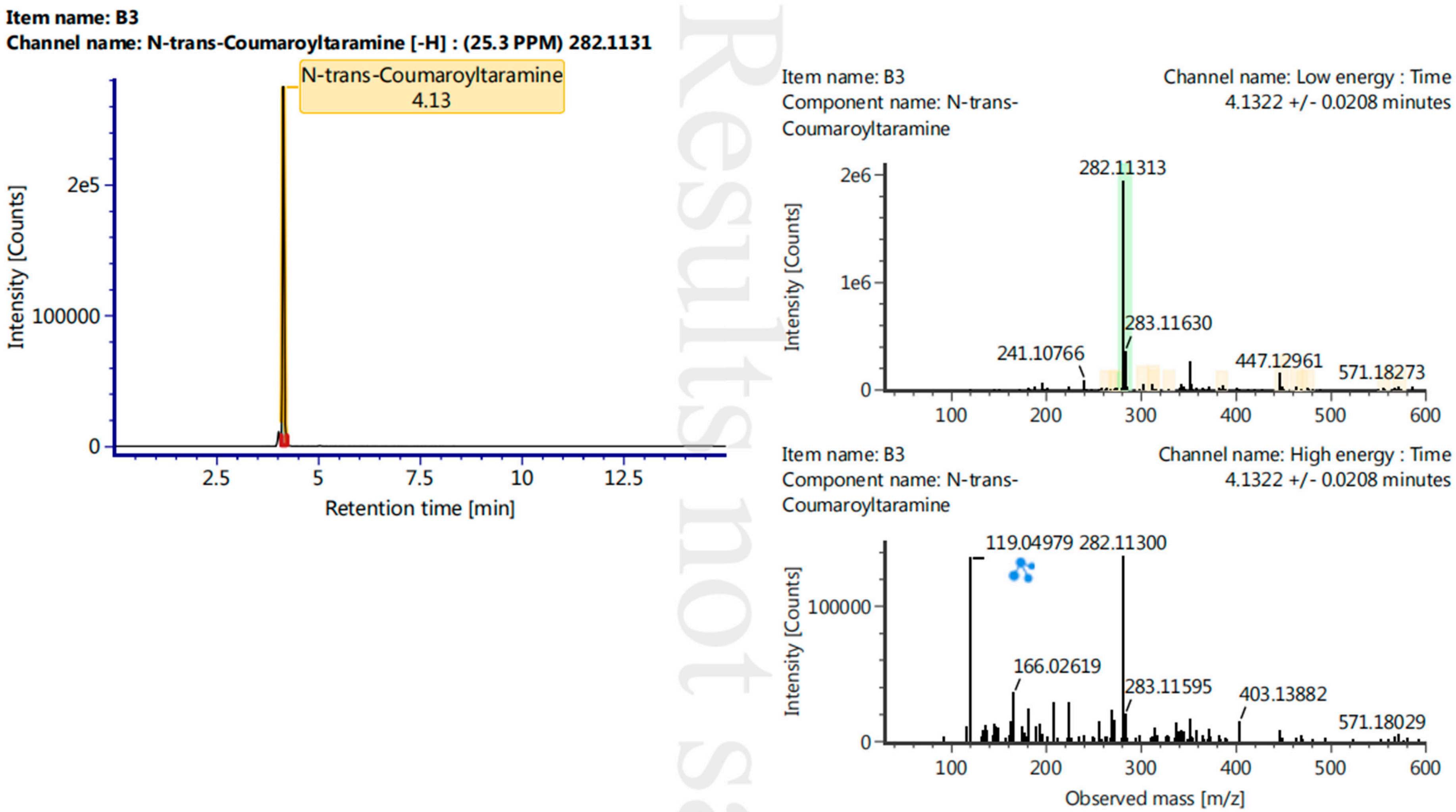

Figure S19. MS/MS spectrum of N-trans-Coumaroyltyramine

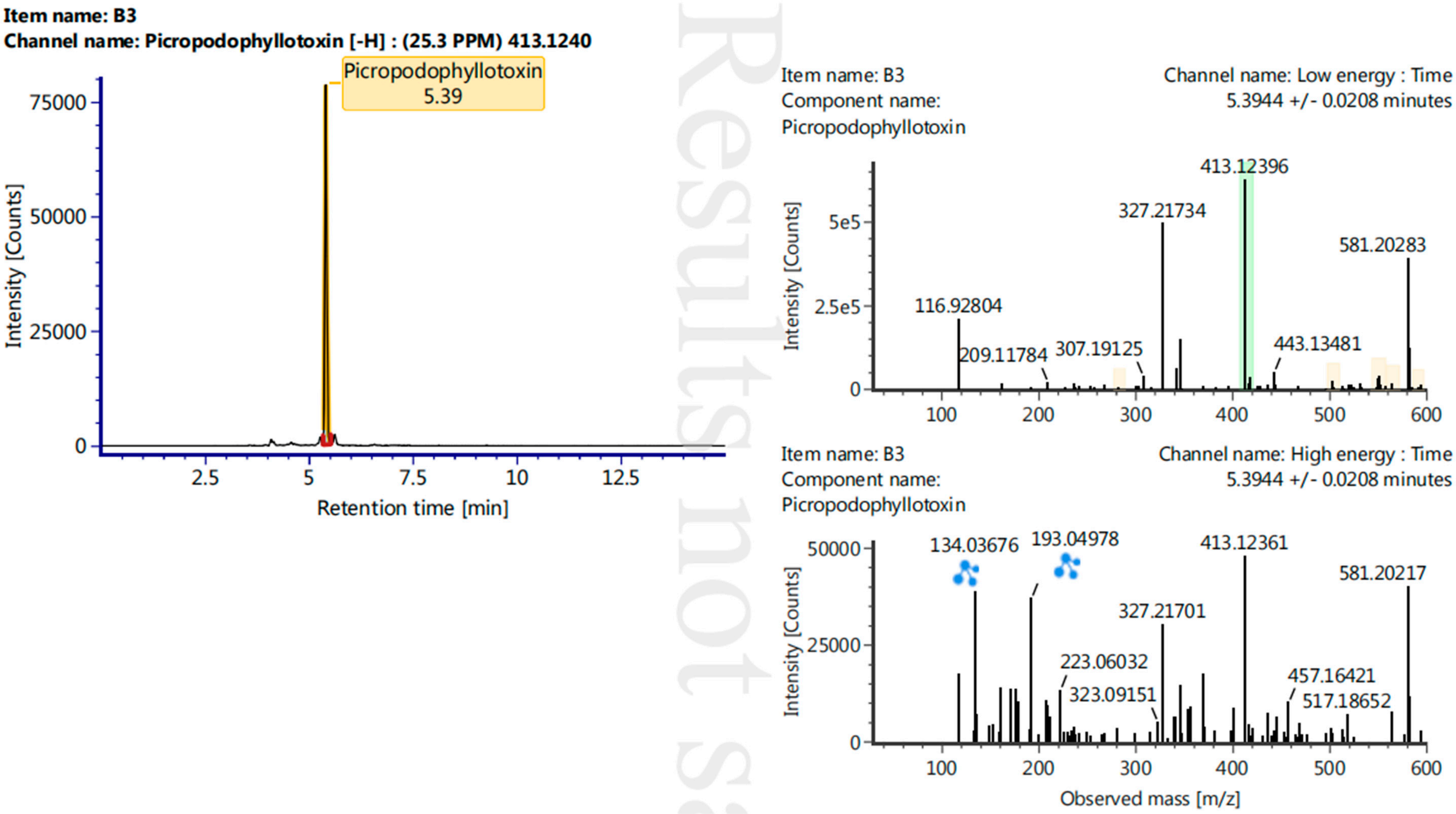

Figure S20. MS/MS spectrum of Picropodophyllotoxin

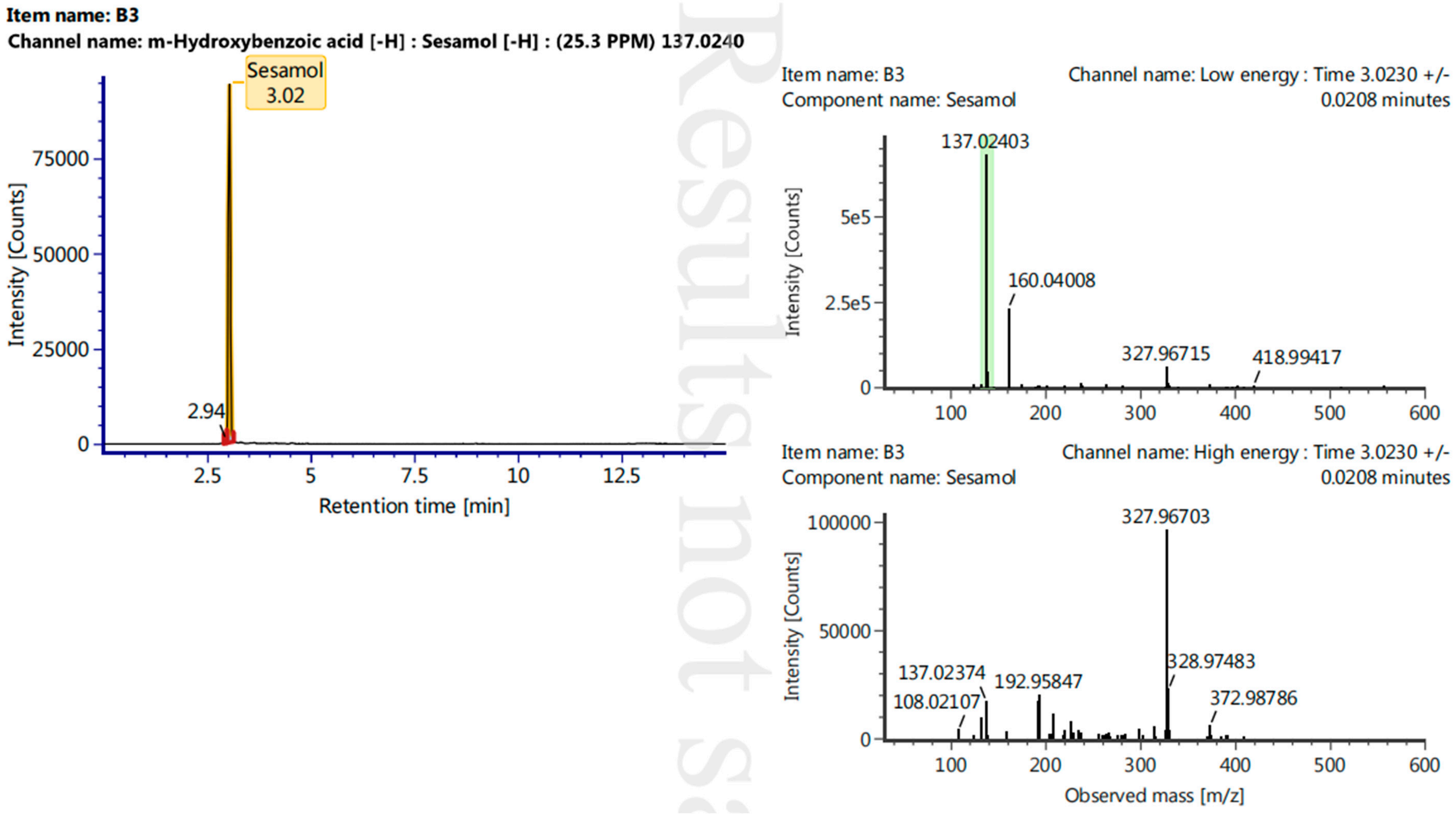

Figure S21. MS/MS spectrum of Sesamol

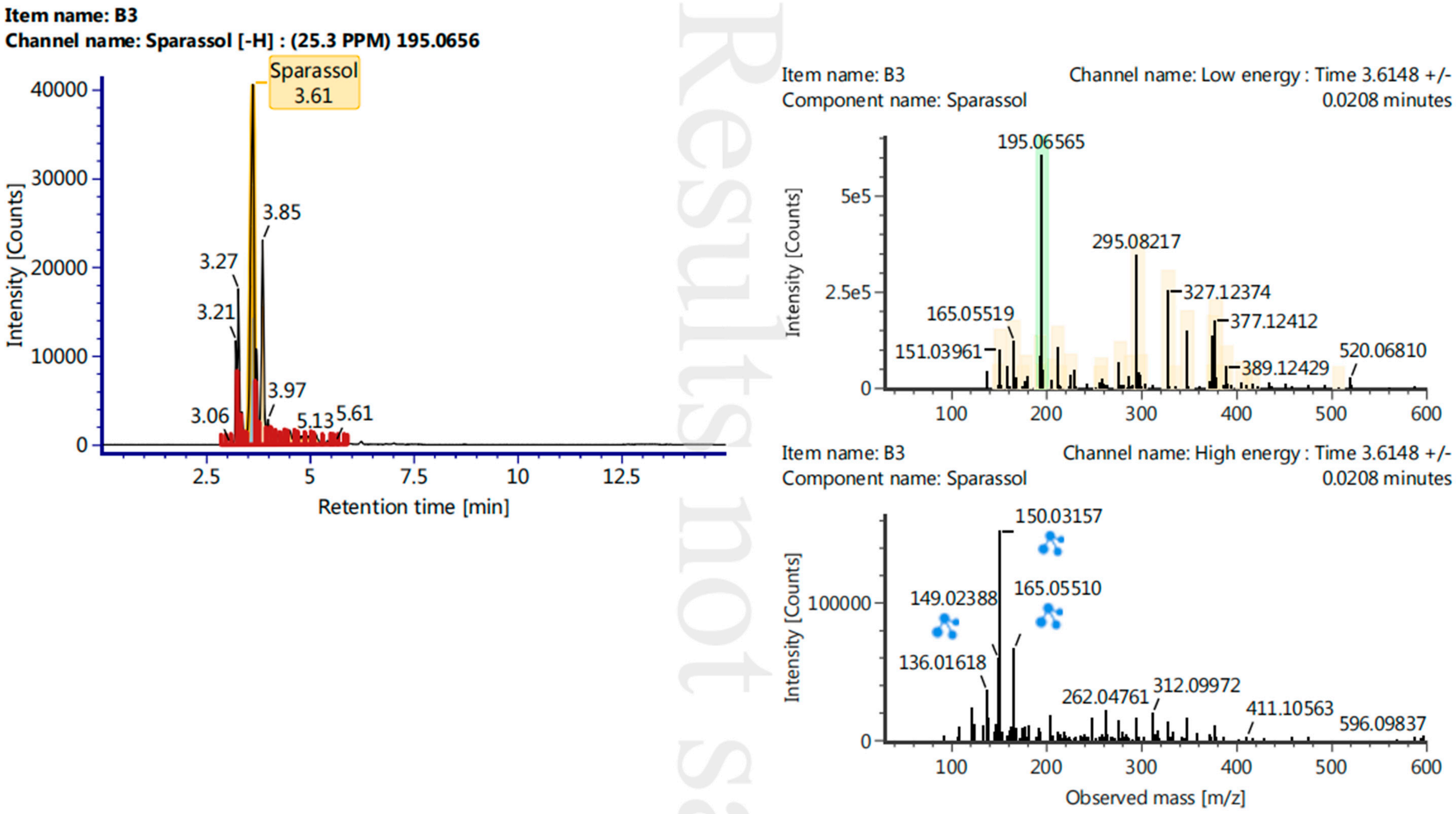

Figure S22. MS/MS spectrum of Sparassol

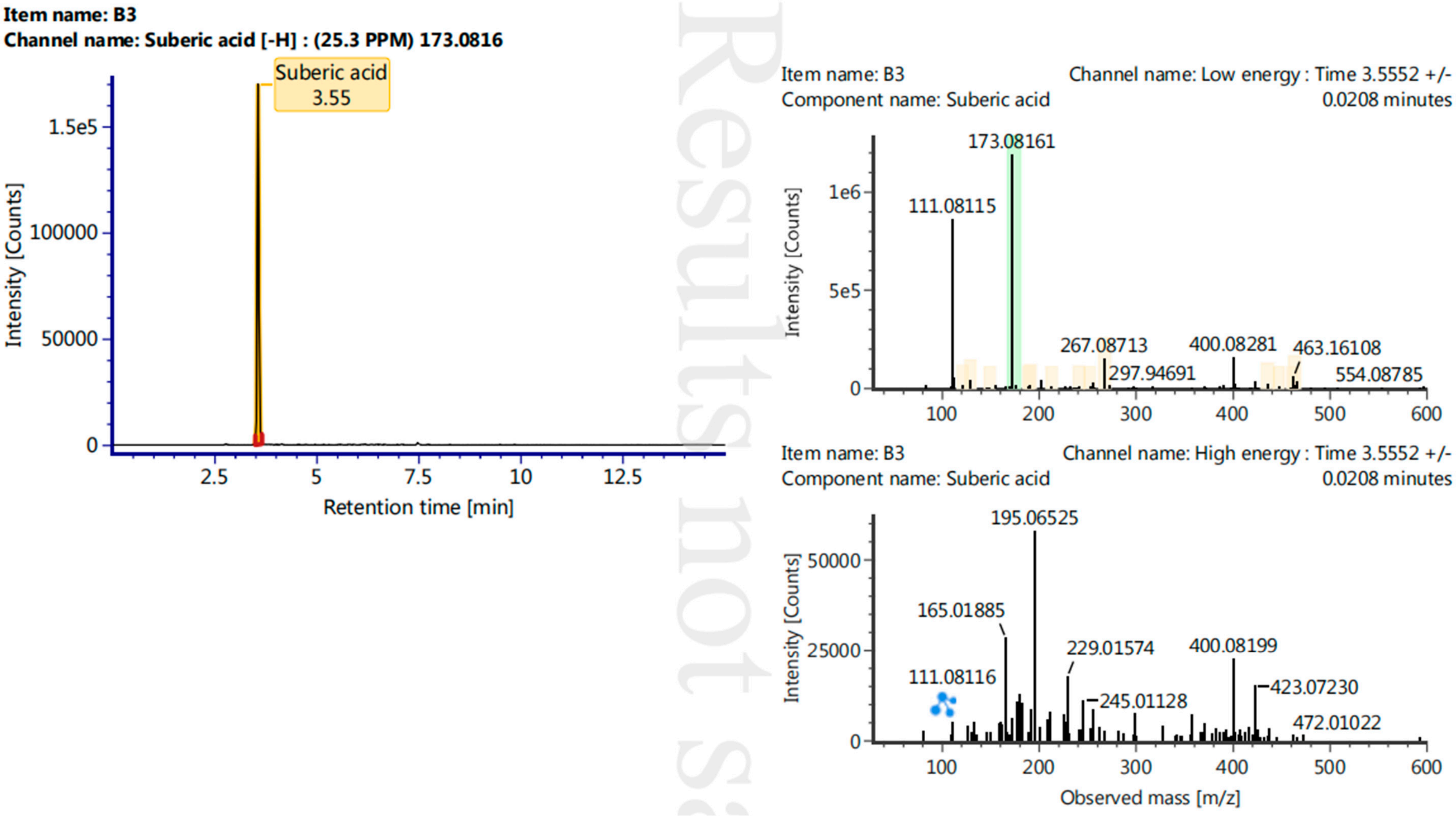

Figure S23. MS/MS spectrum of Suberic acid

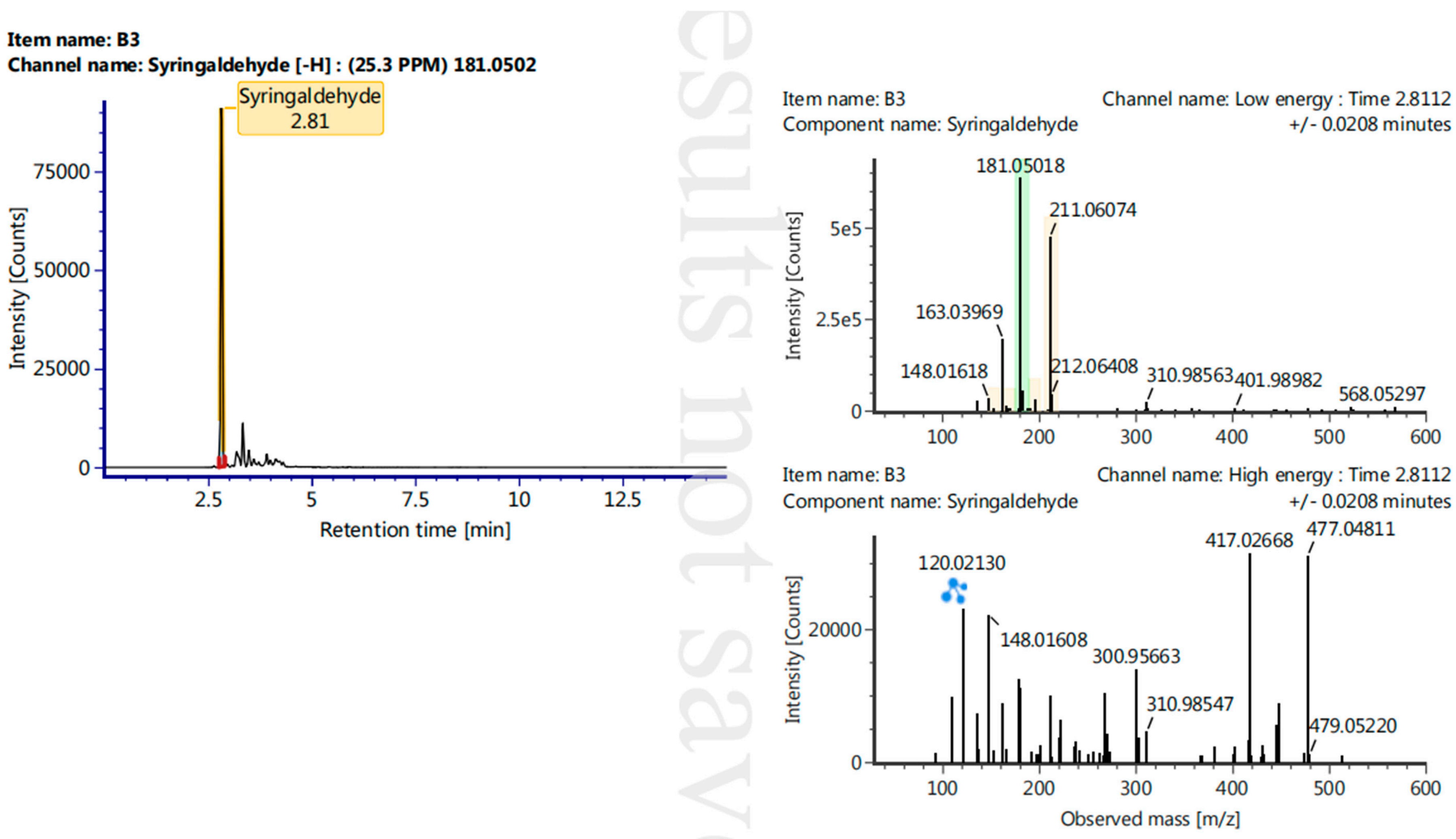

esults not sav

Figure S24. MS/MS spectrum of Syringaldehyde

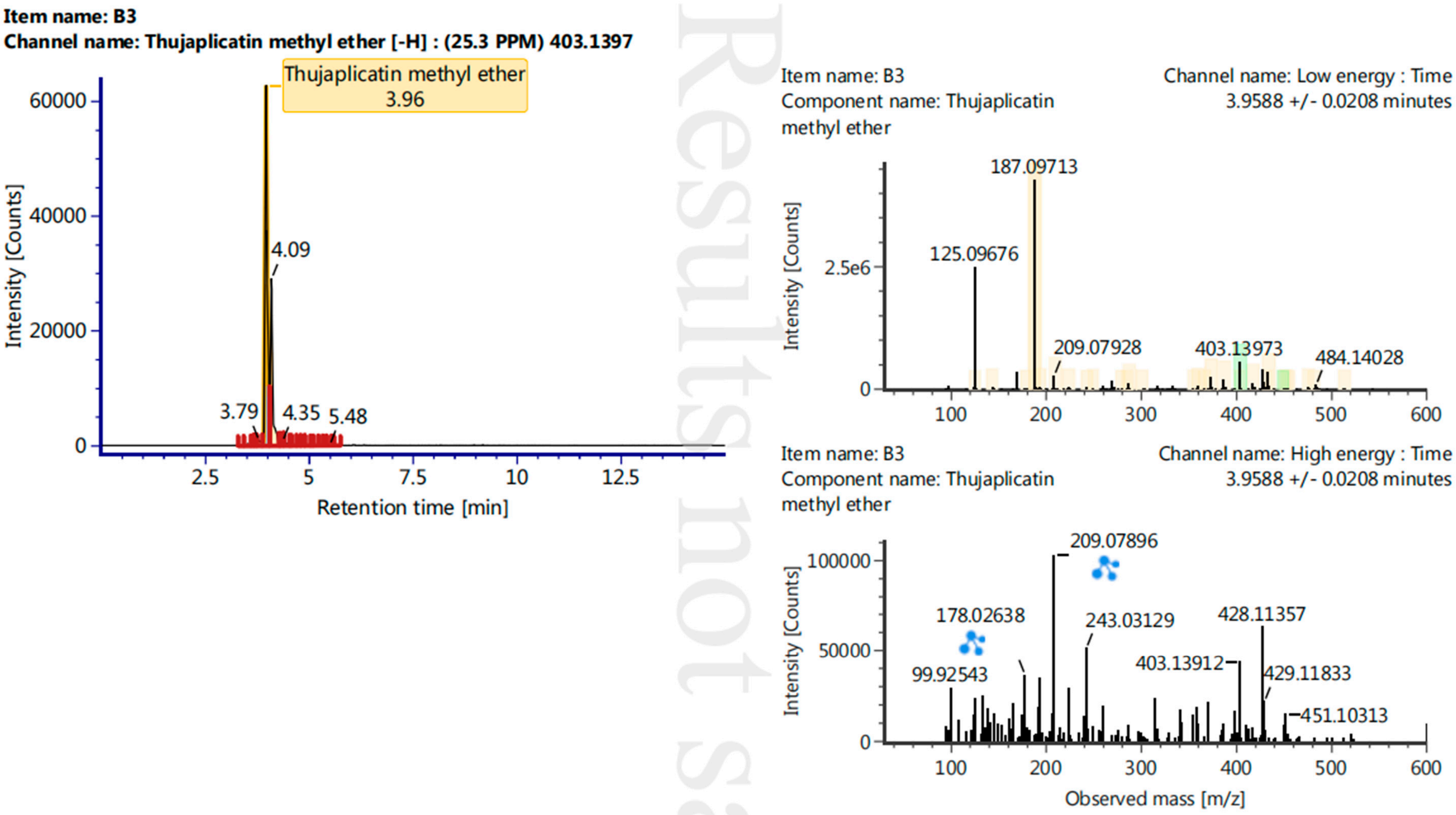

Figure S25. MS/MS spectrum of Thujaplicatin methyl ether

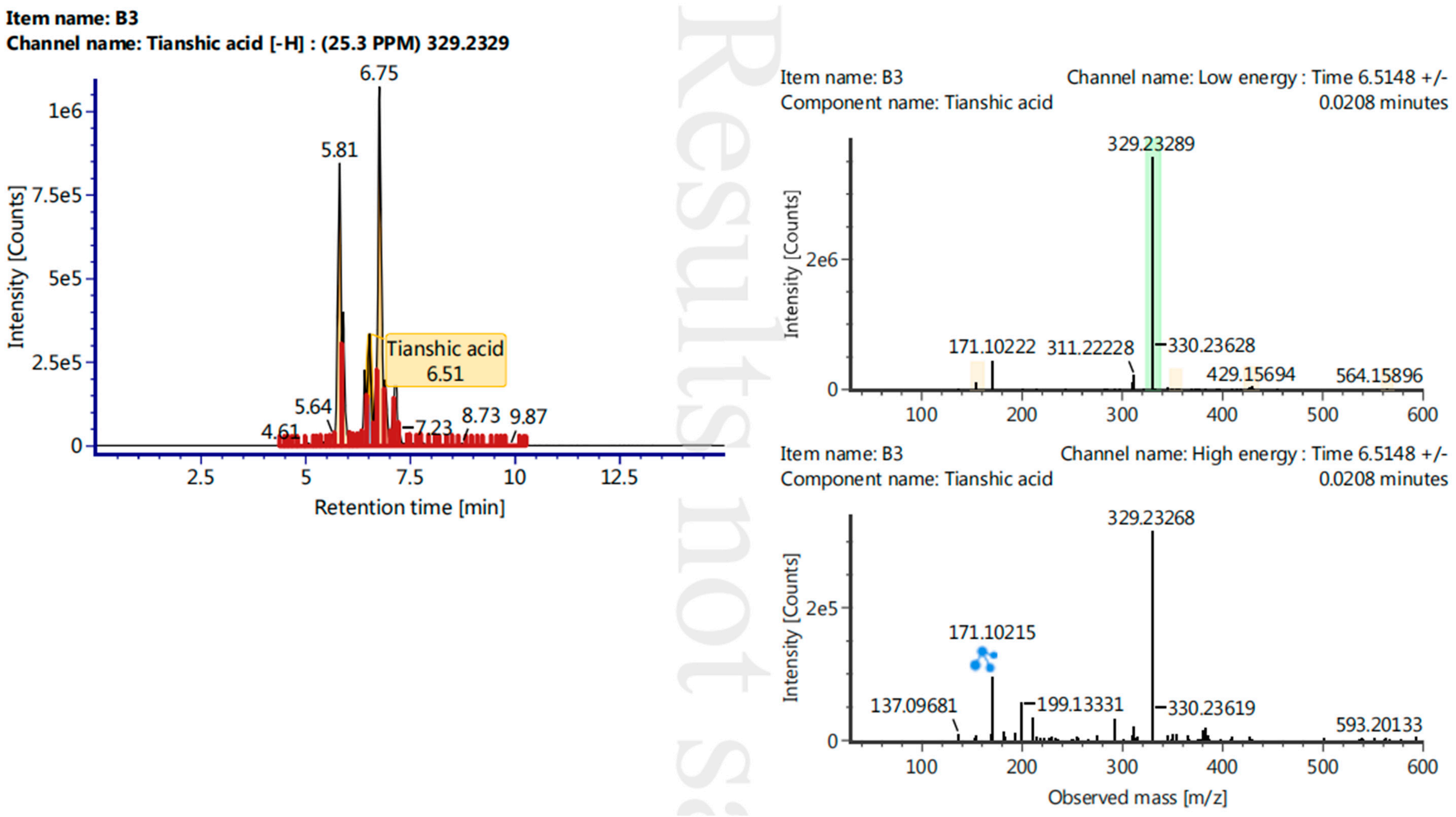

Figure S26. MS/MS spectrum of Tianshic acid

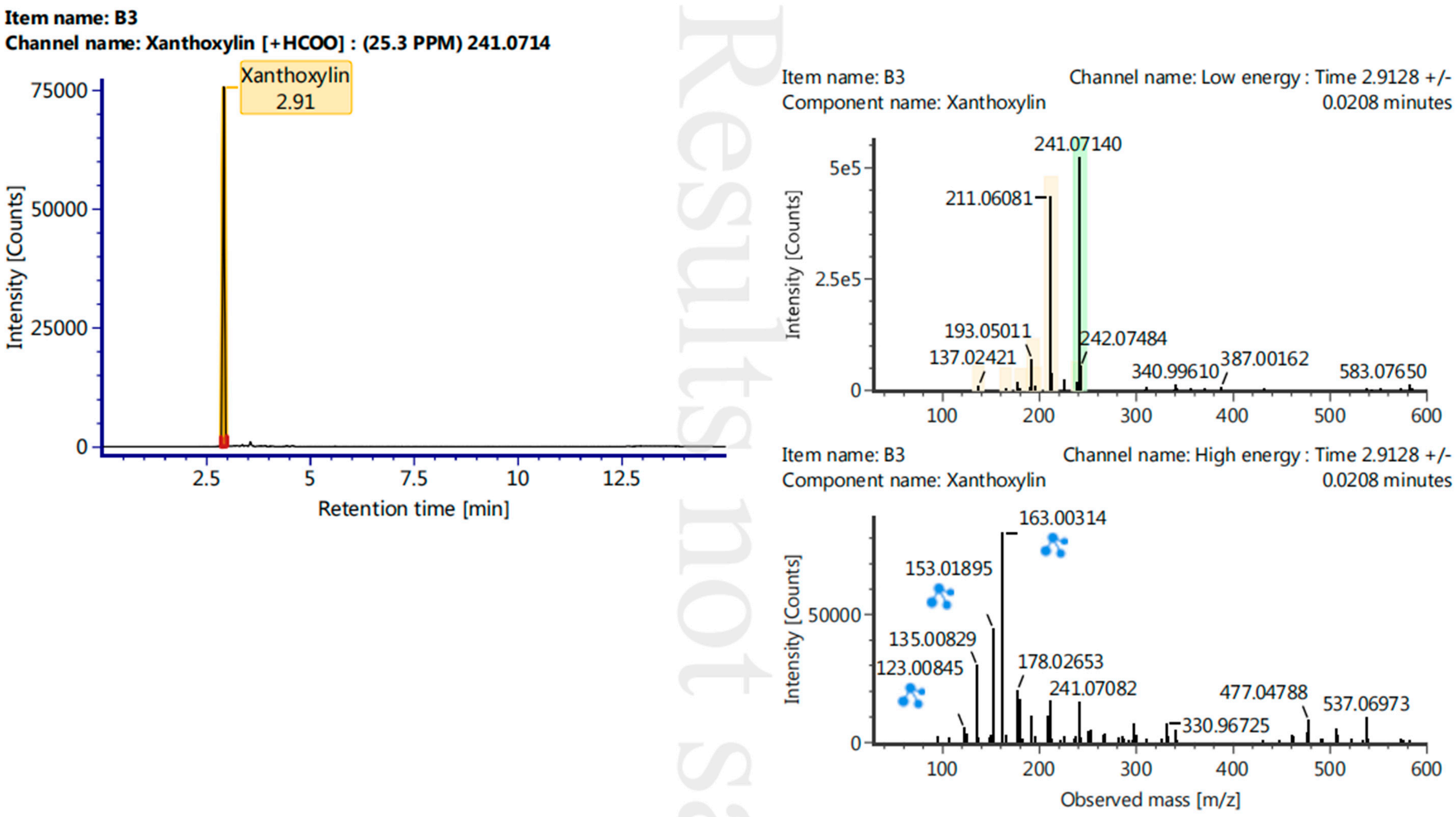

Figure S27. MS/MS spectrum of Xanthoxylin

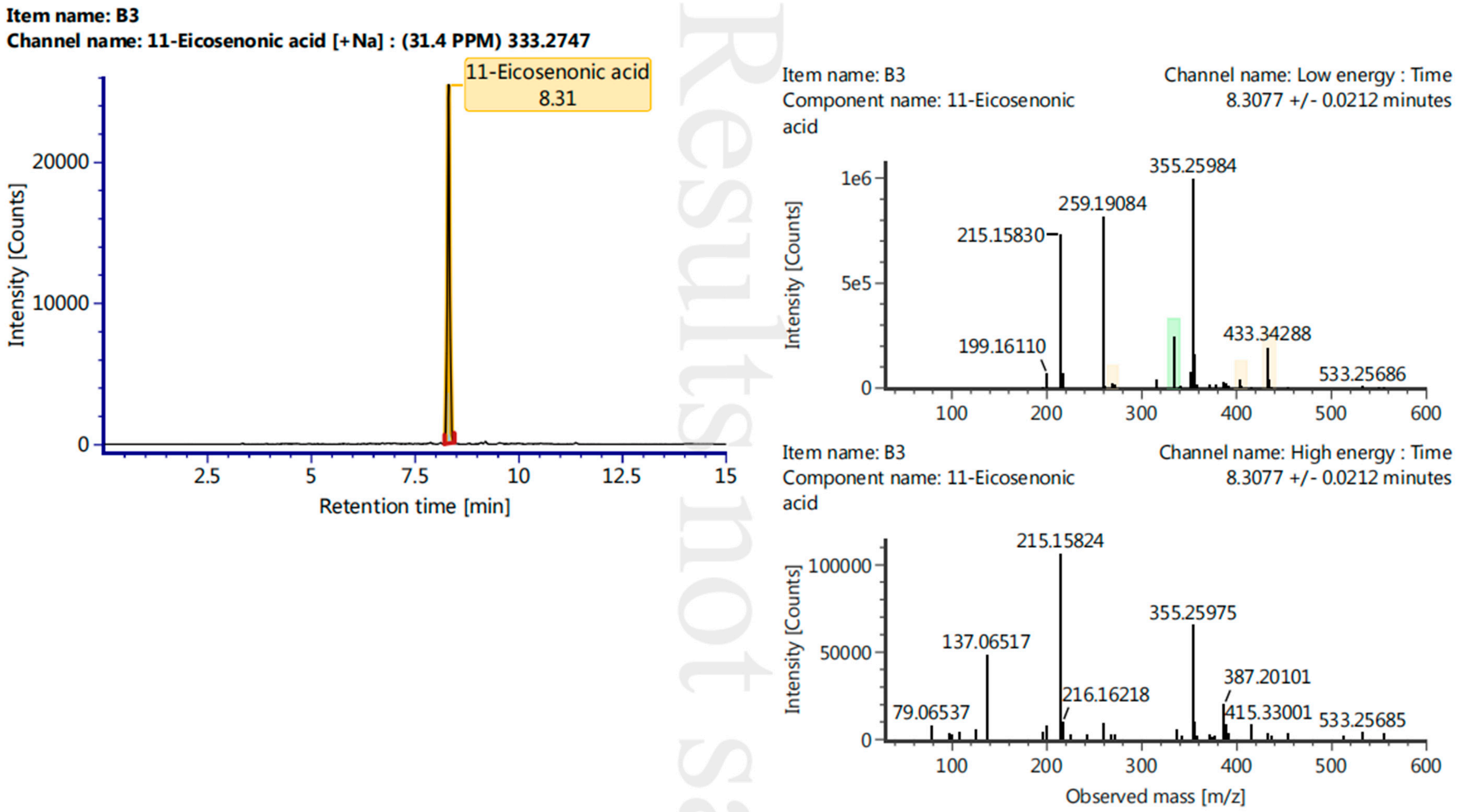

Figure S28. MS/MS spectrum of 11-Eicosenonic acid

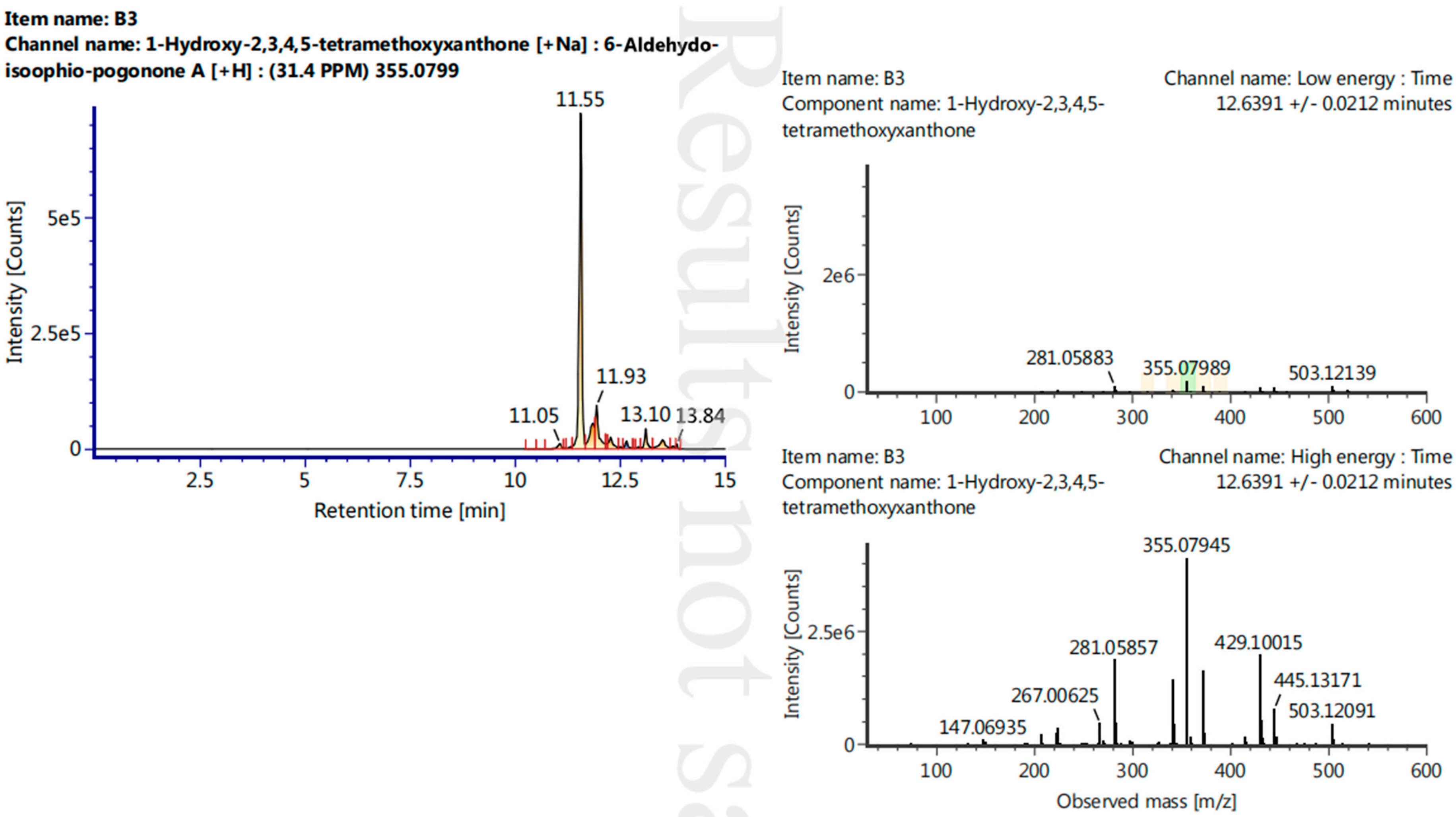

Results not Sa

Figure S29. MS/MS spectrum of 1-Hydroxy-2,3,4,5- tetramethoxyxanthone

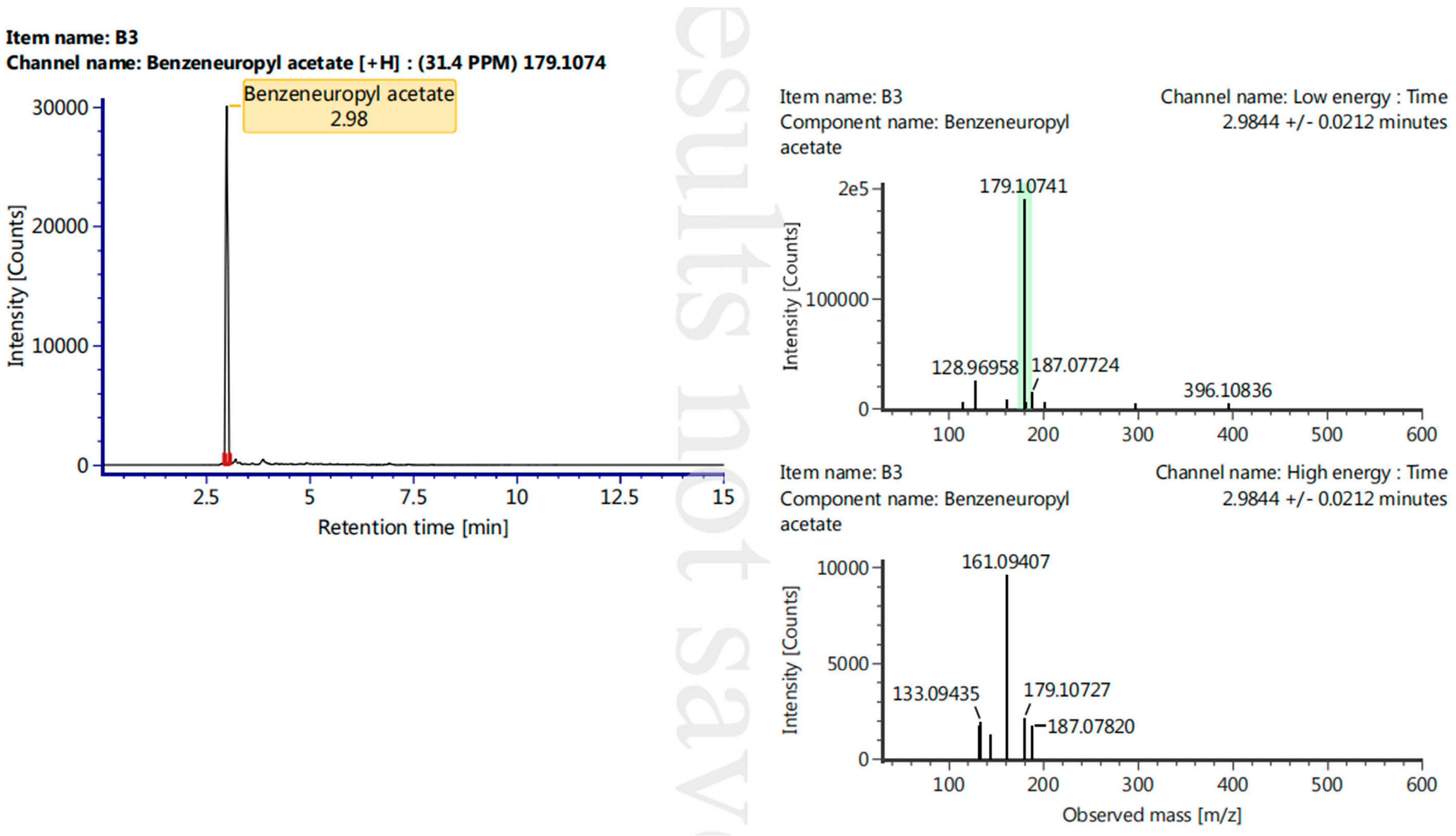

Figure S30. MS/MS spectrum of Benzeneuropyl acetate

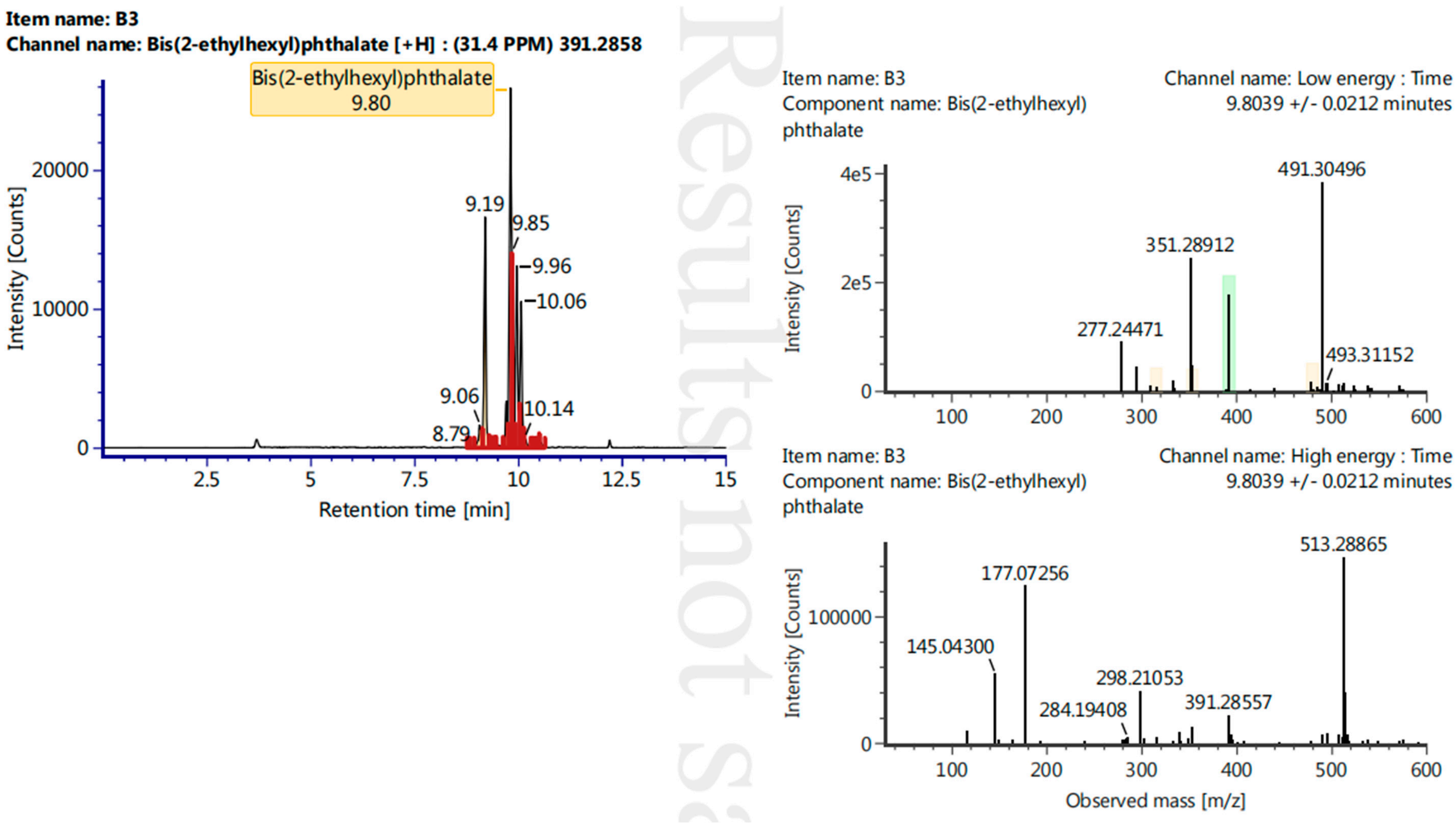

Figure S31. MS/MS spectrum of Bis(2-ethylhexyl) phthalate

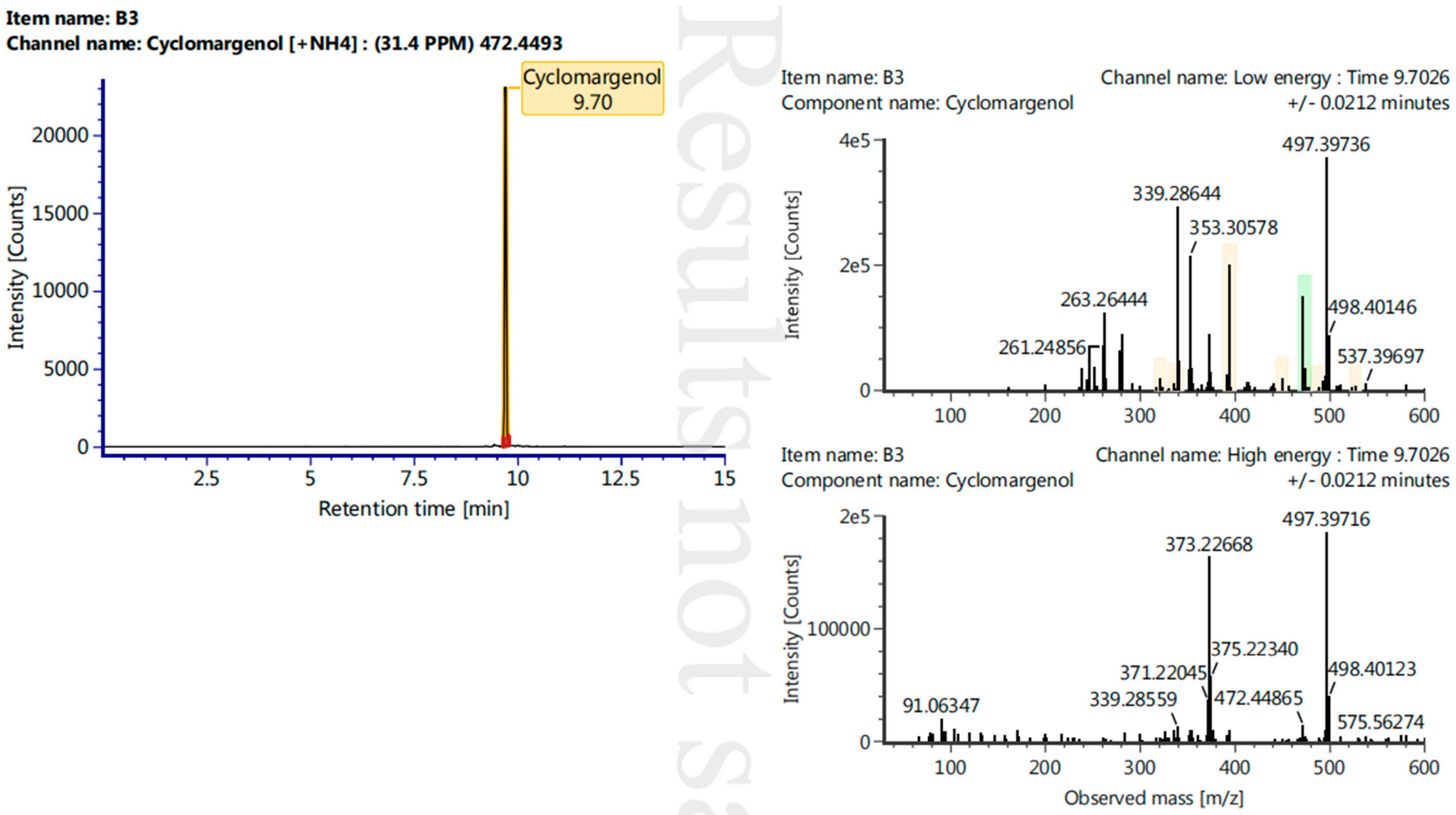

Figure S32. MS/MS spectrum of Cyclomargenol

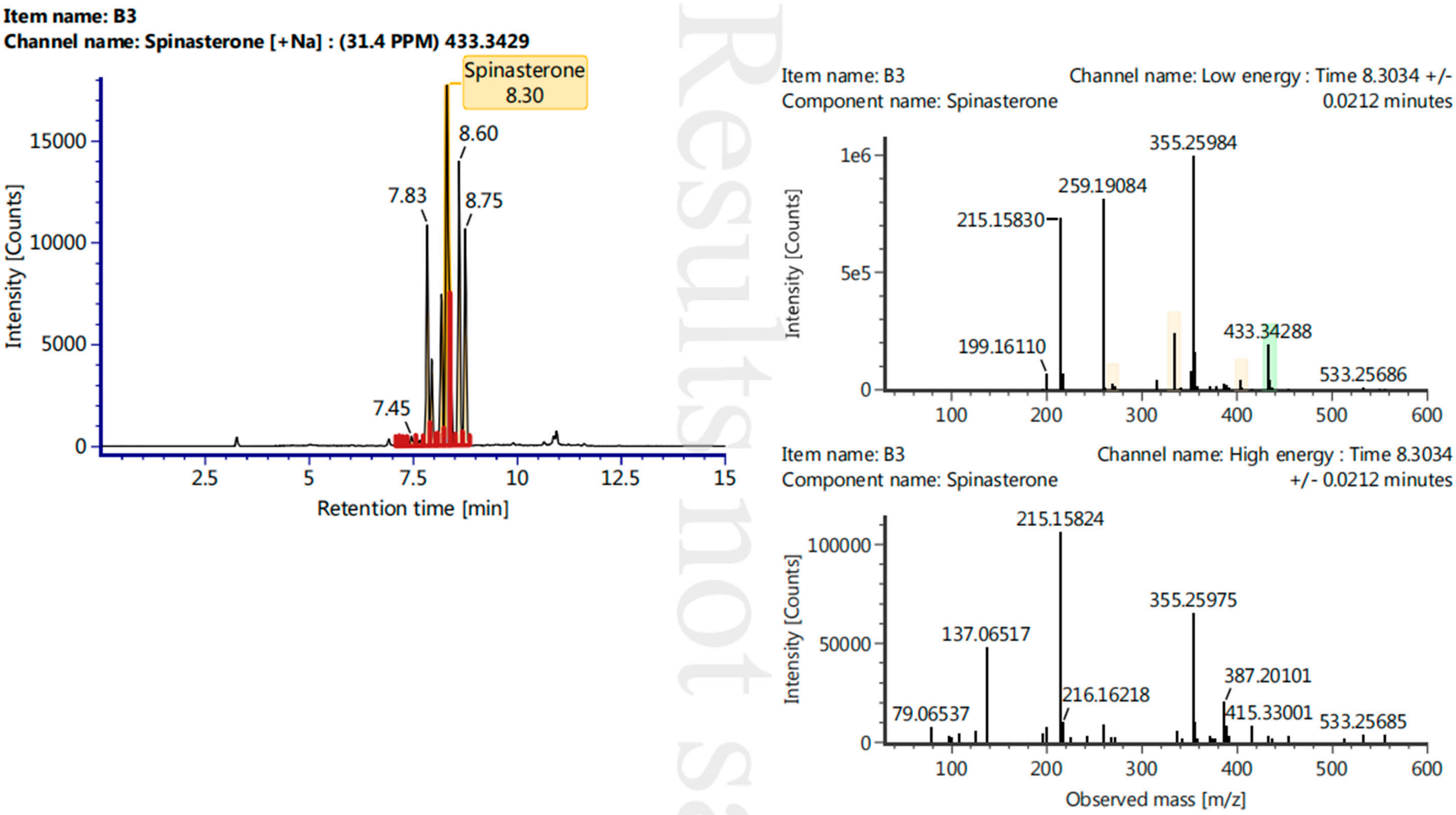

Figure S33. MS/MS spectrum of Spinasterone

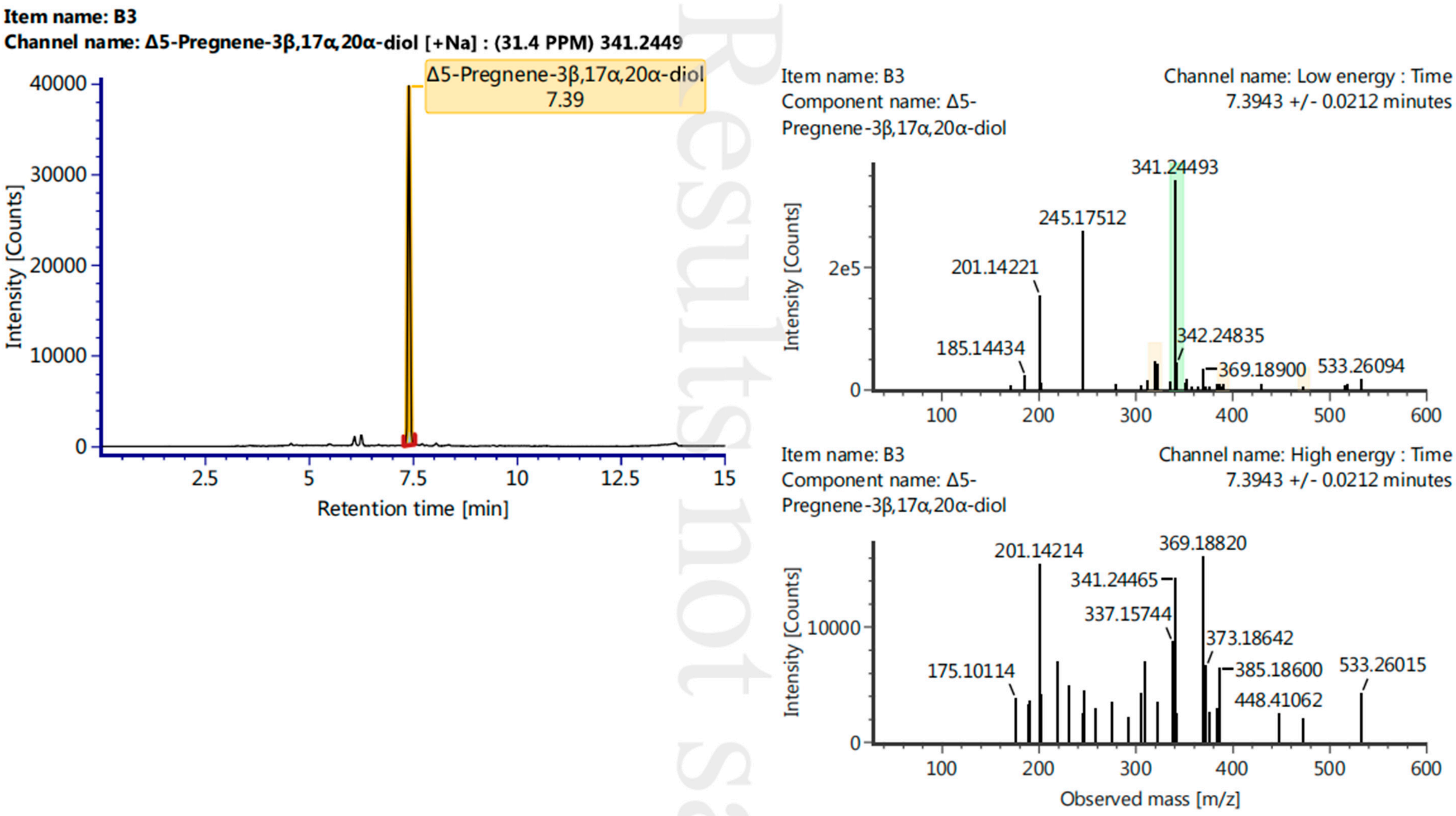

Figure S34. MS/MS spectrum of Δ5-Pregnene-3β,17α,20α-diol

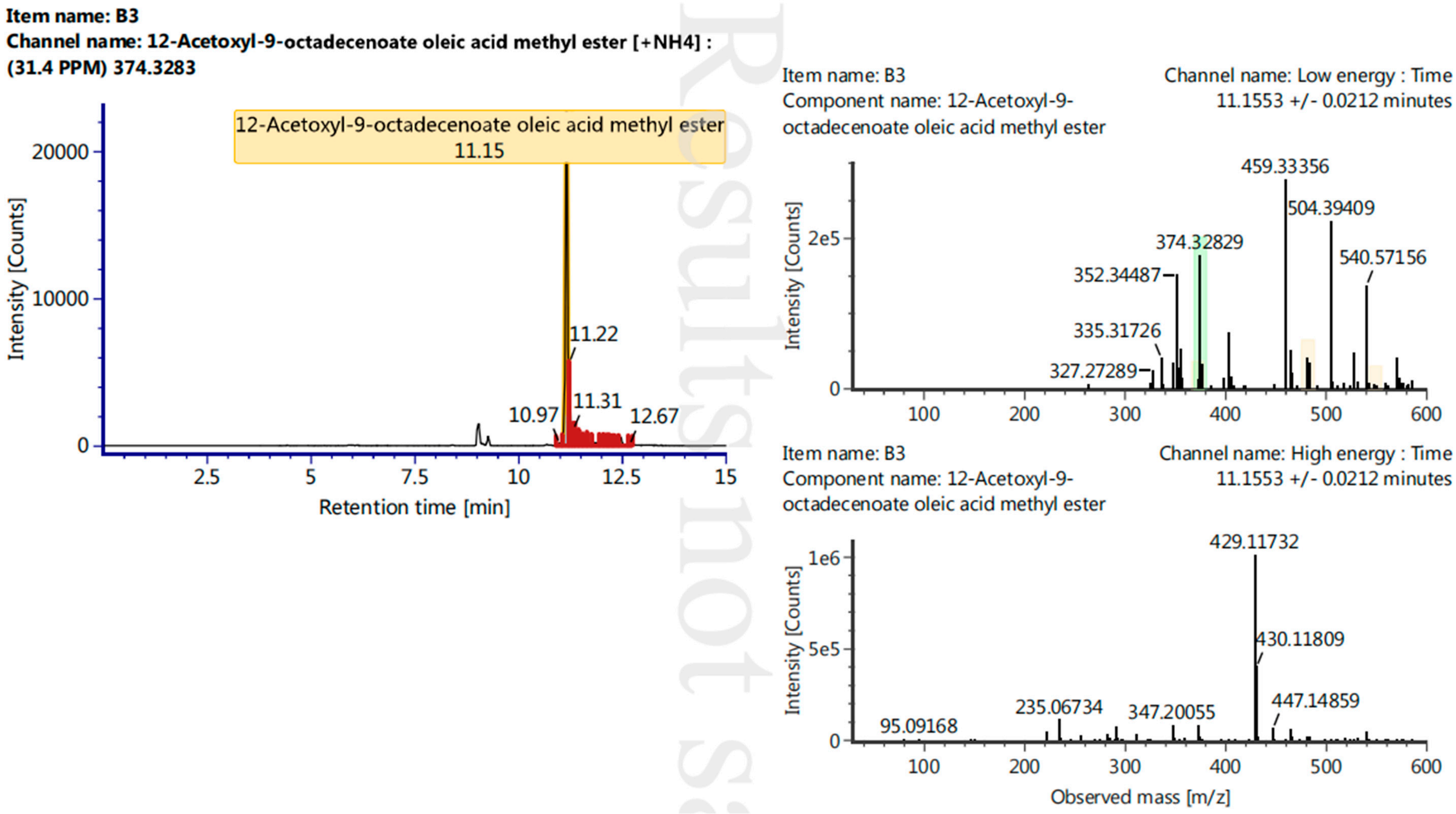

Figure S35. MS/MS spectrum of 12-Acetoxyl-9- octadecenoate oleic acid methyl ester

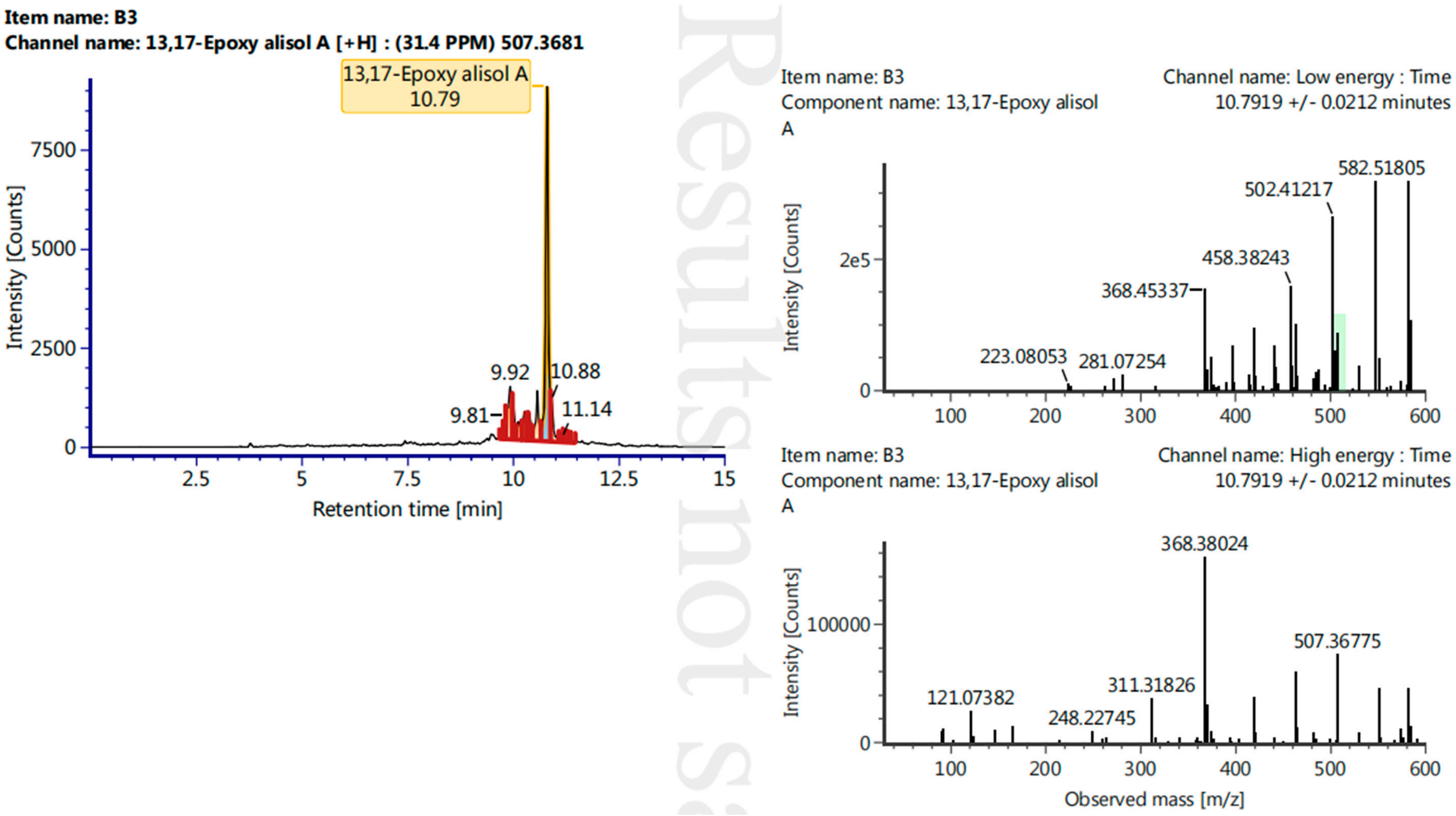

Figure S36. MS/MS spectrum of 13,17-Epoxy alisol A

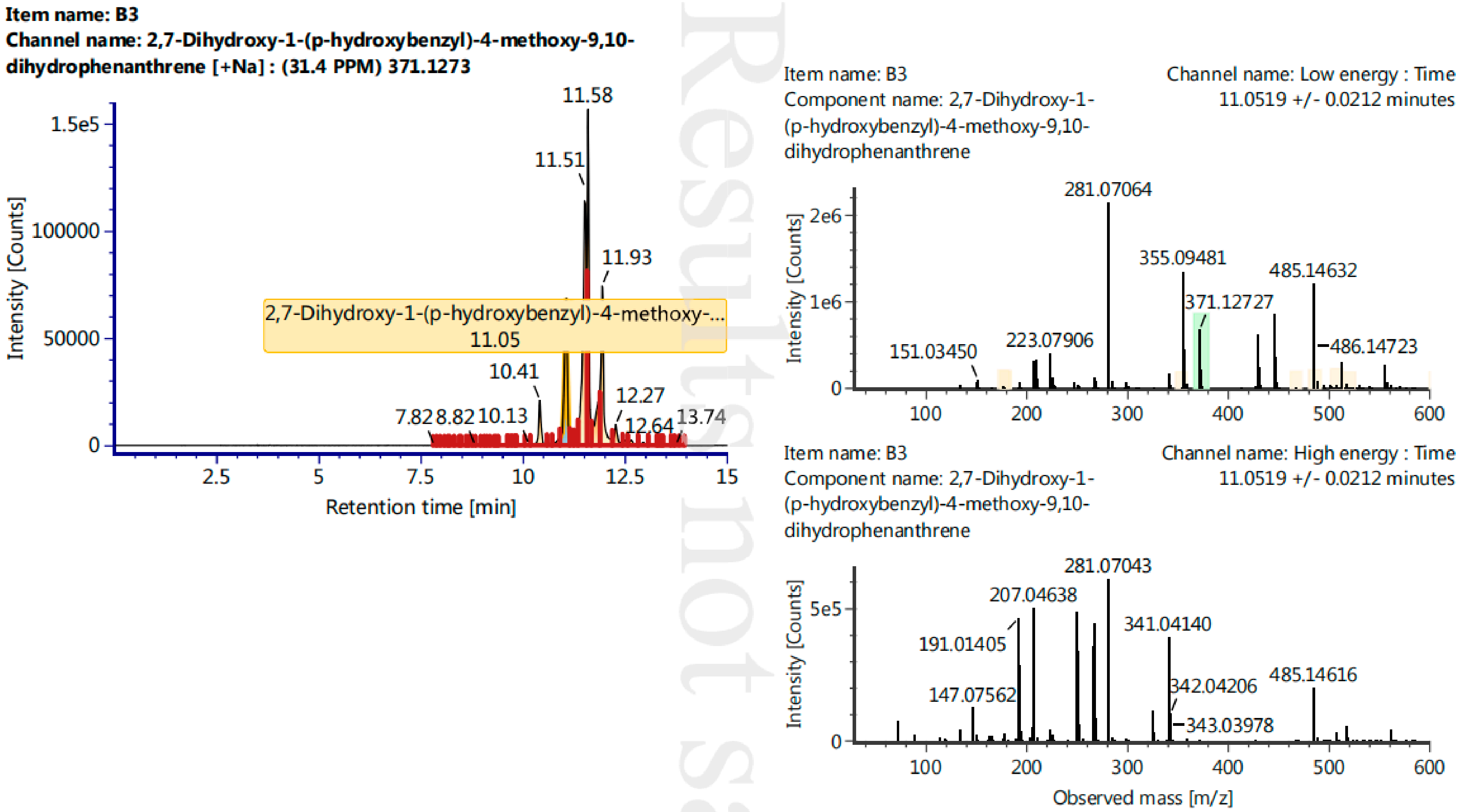

Figure S37. MS/MS spectrum of 2,7-Dihydroxy-1-(p-hydroxybenzyl)-4-methoxy-9,10- dihydrophenanthrene

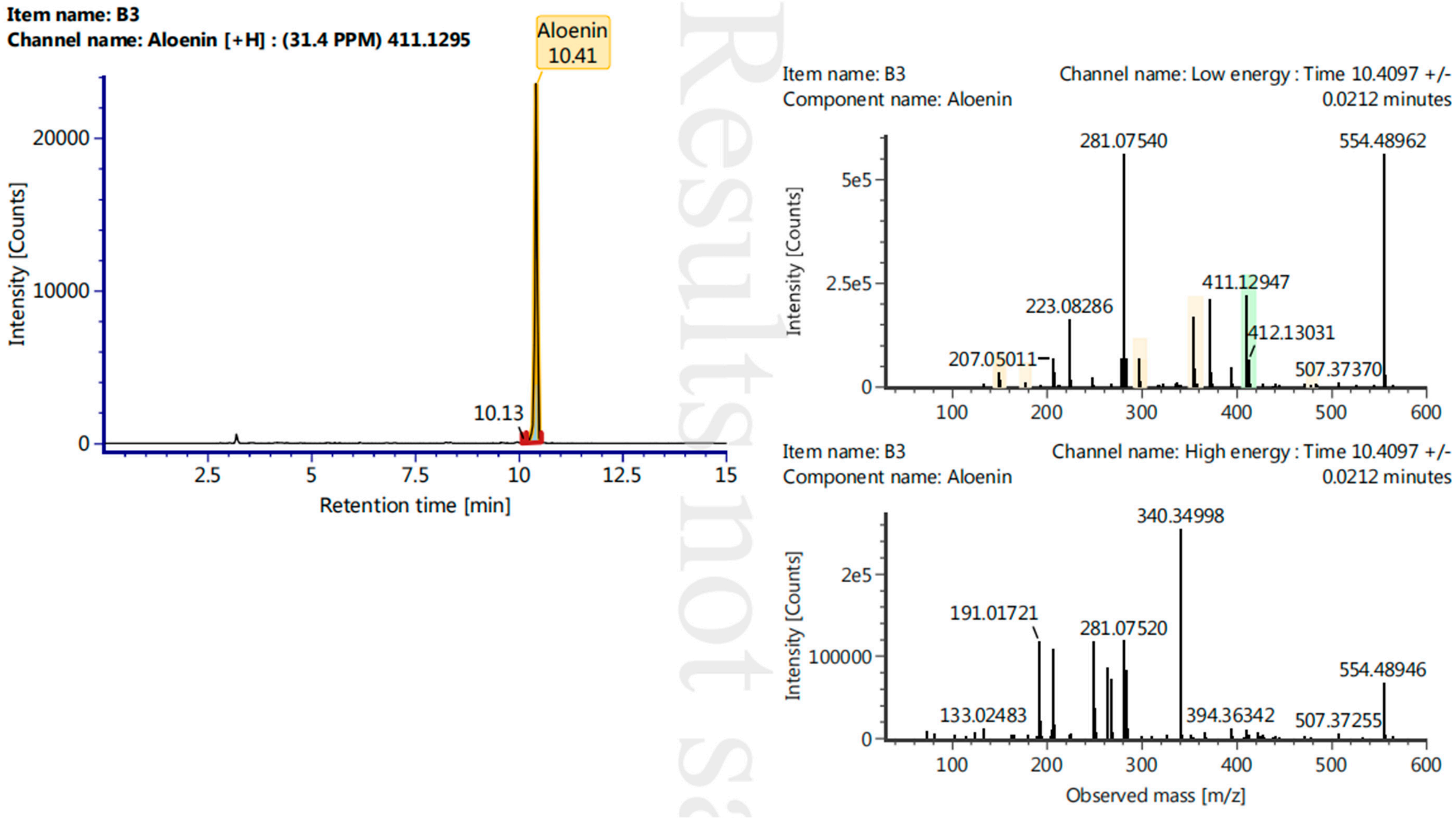

Figure S38. MS/MS spectrum of Aloenin

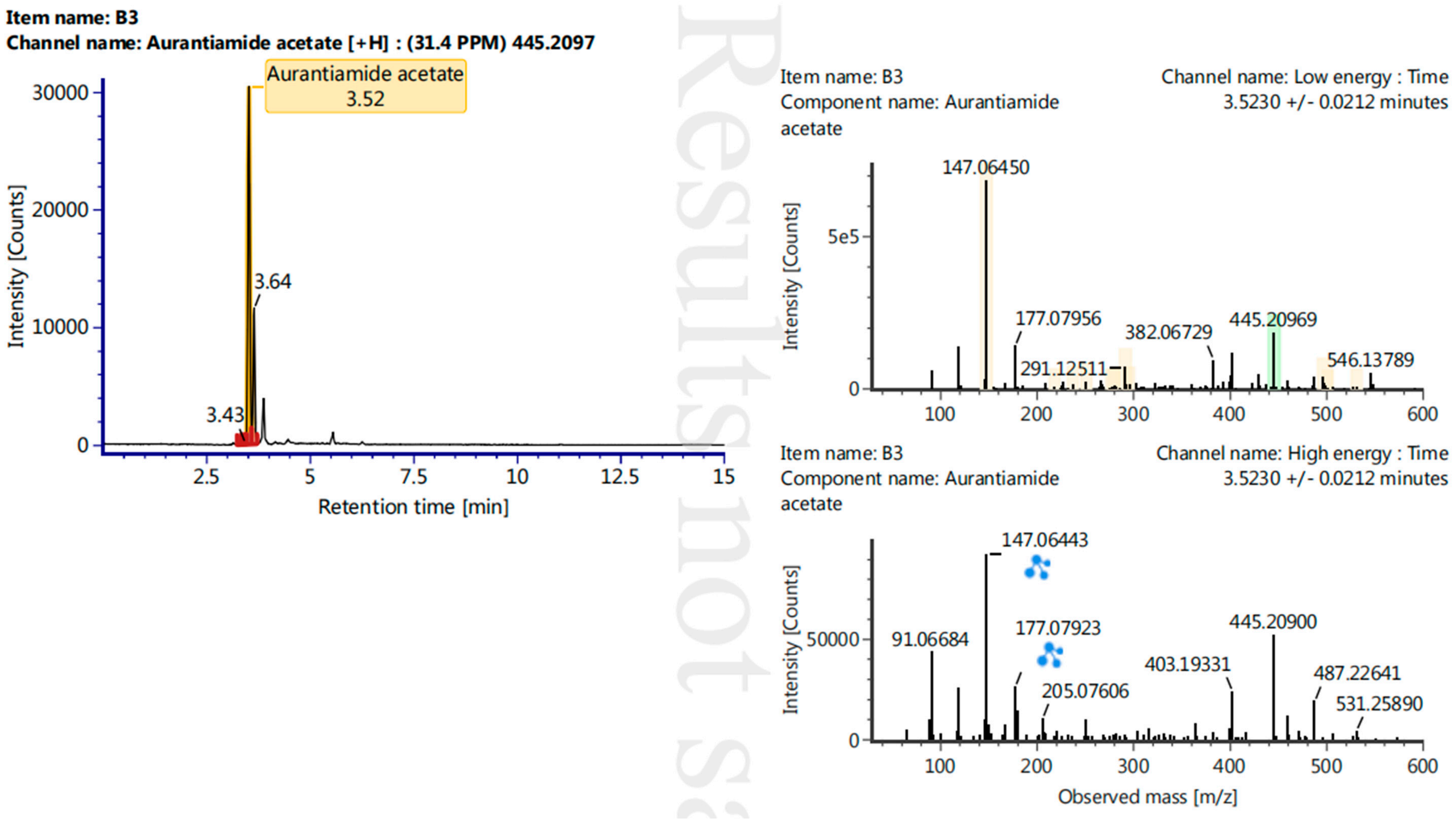

Figure S39. MS/MS spectrum of Aurantiamide acetate

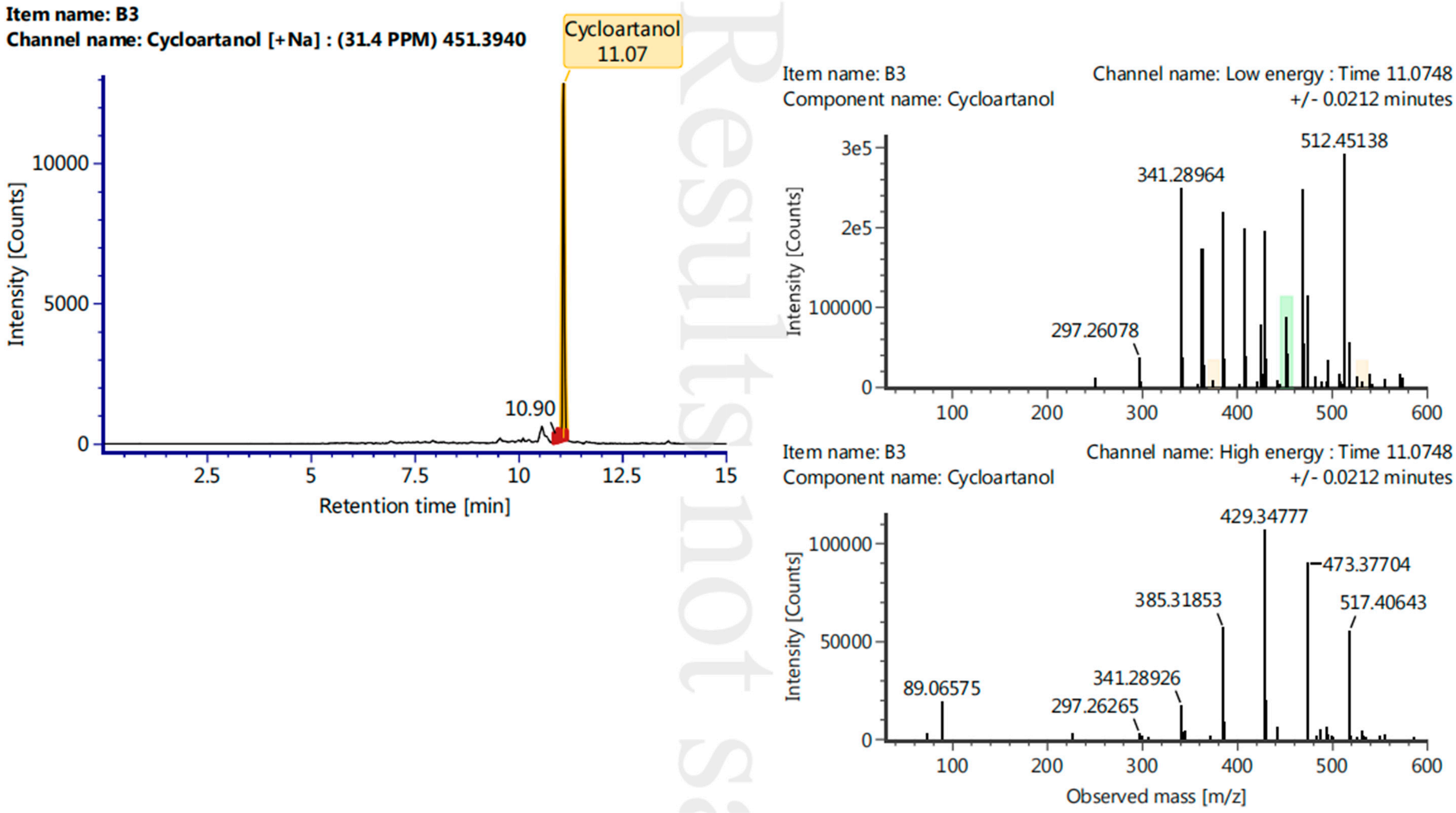

Results not s

Figure S40. MS/MS spectrum of Cycloartanol

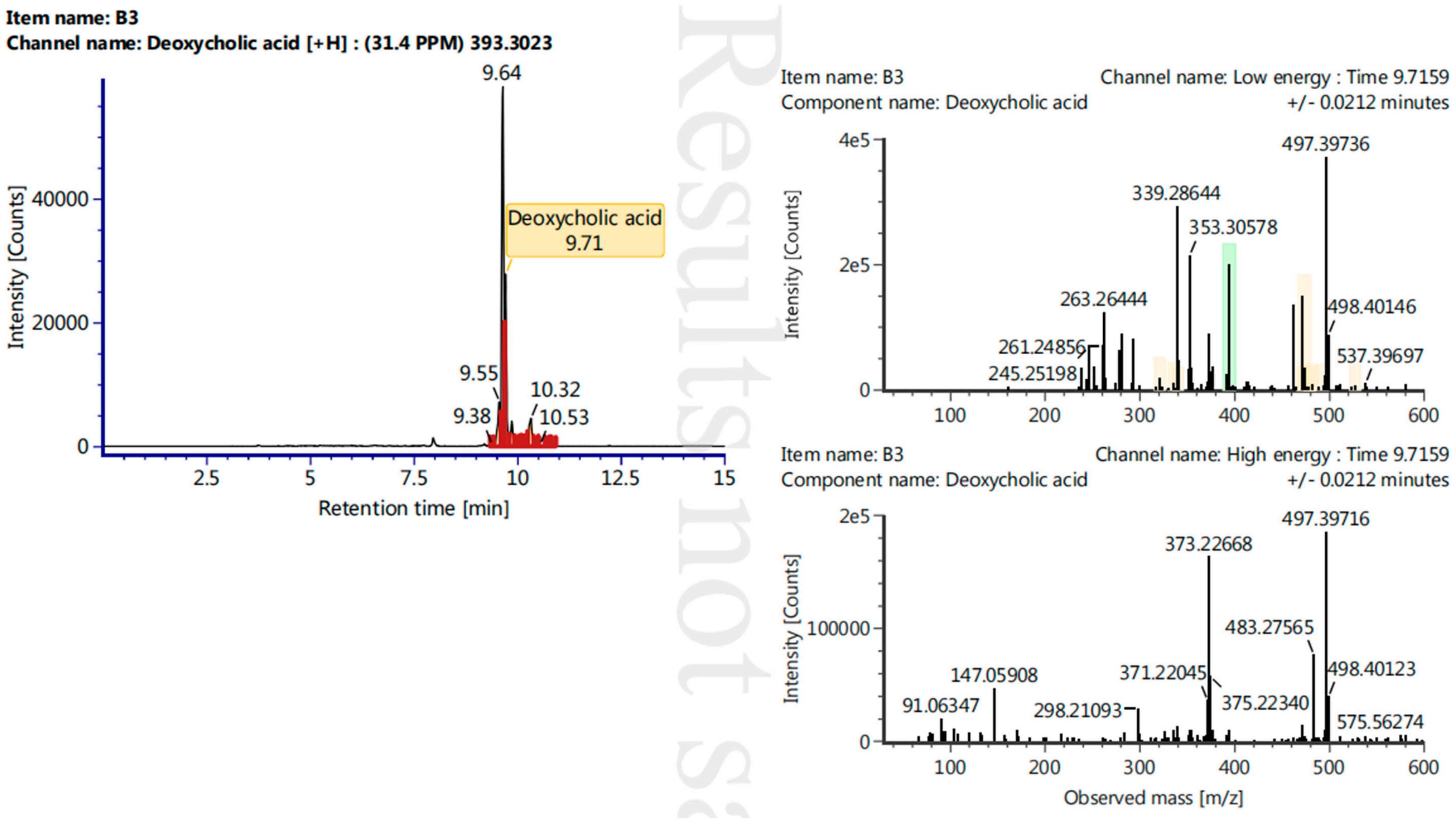

Figure S41. MS/MS spectrum of Deoxycholic acid

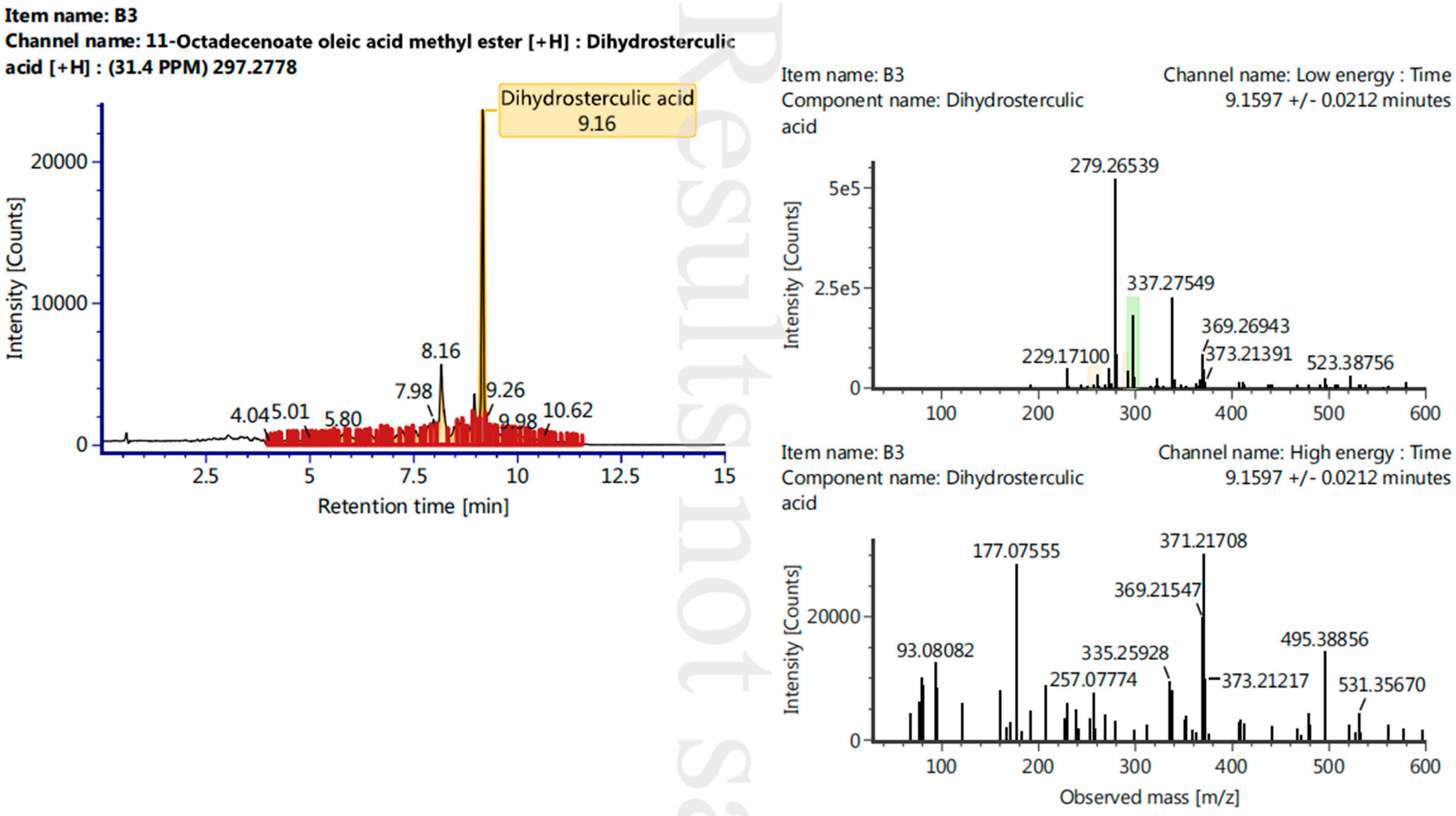

Figure S42. MS/MS spectrum of Dihydrosterculic acid

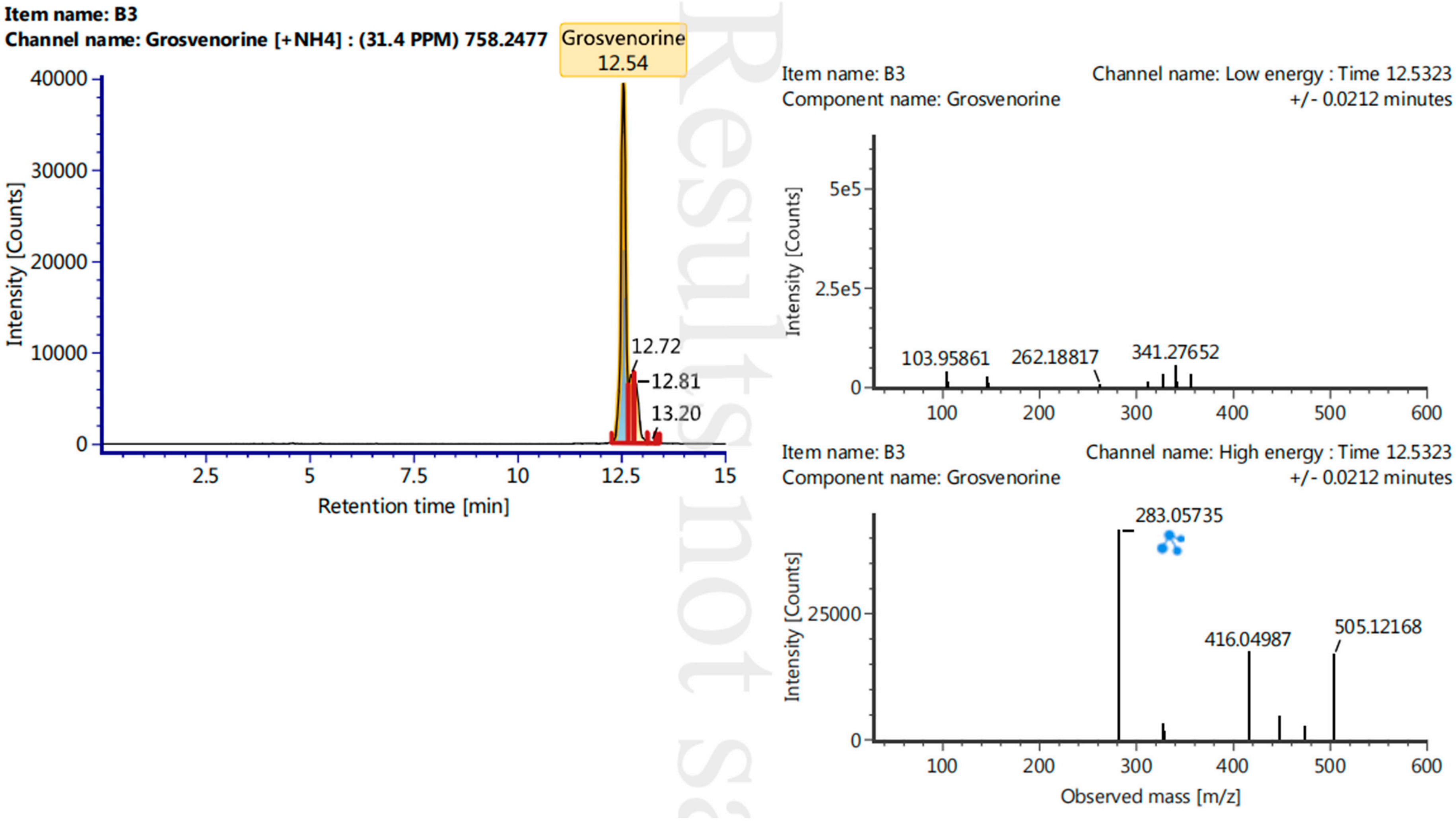

Results not Sa

Figure S43. MS/MS spectrum of Grosvenorine

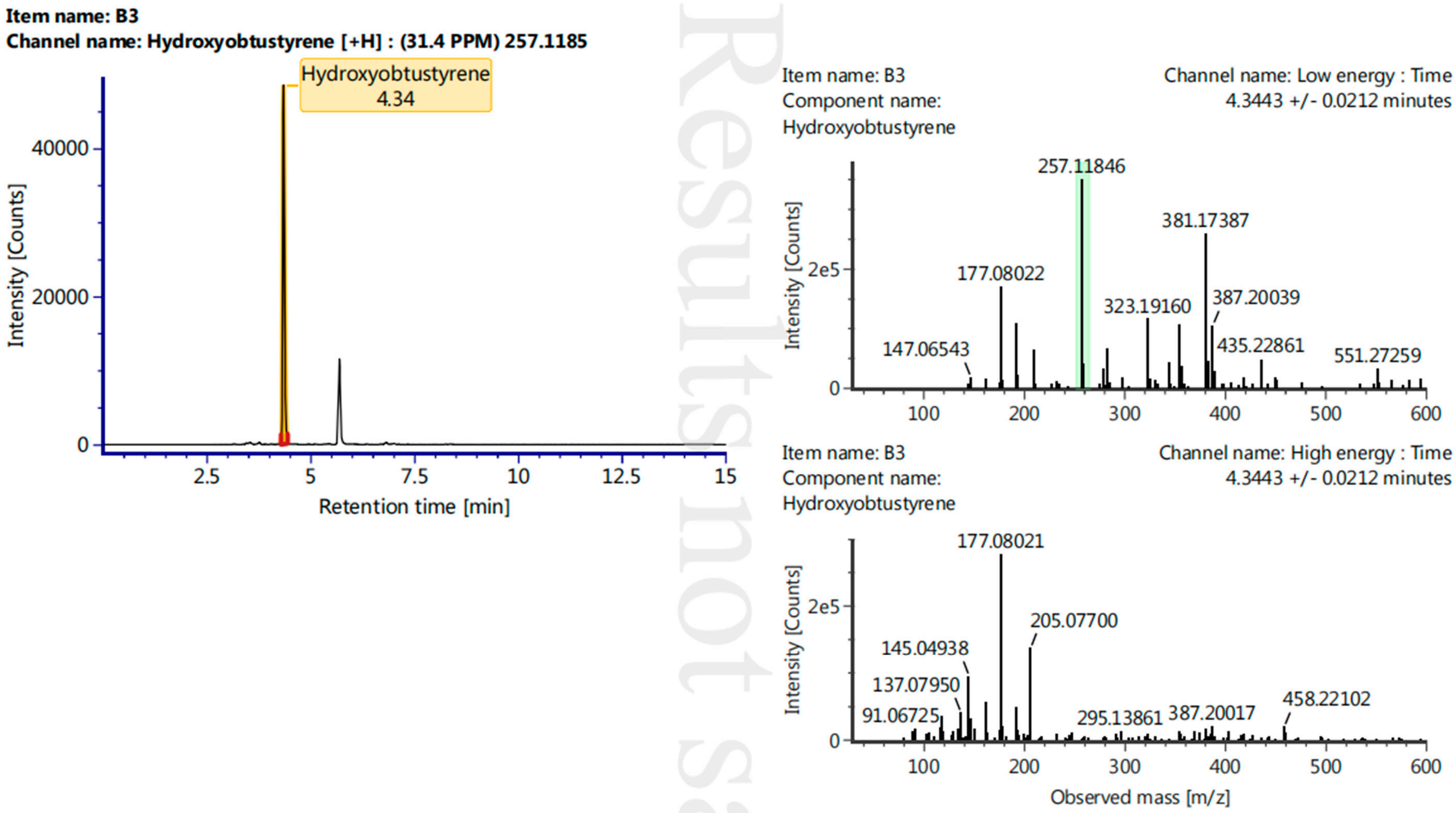

Figure S44. MS/MS spectrum of Hydroxyobtustyrene

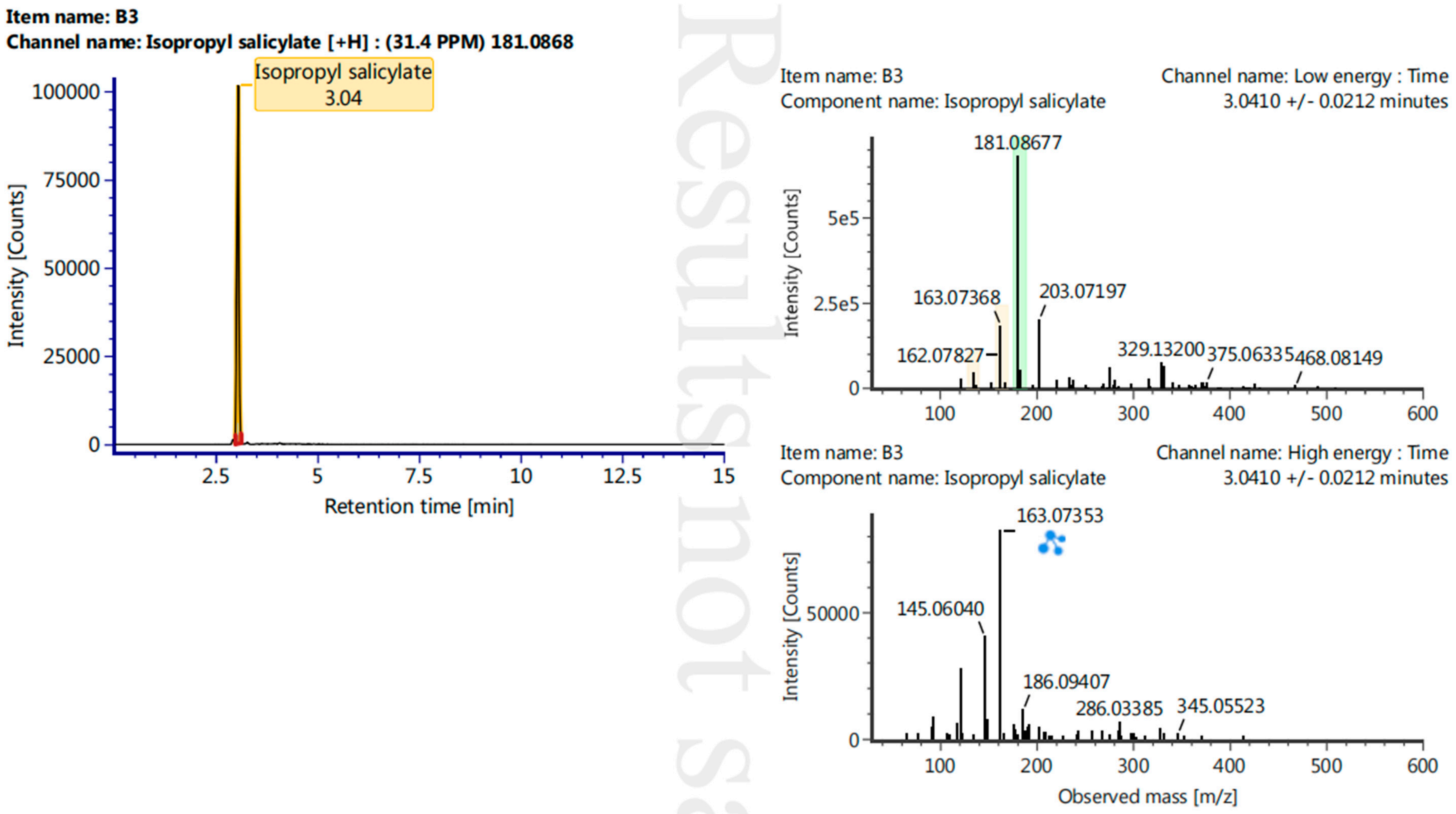

Figure S45. MS/MS spectrum of Isopropyl salicylate

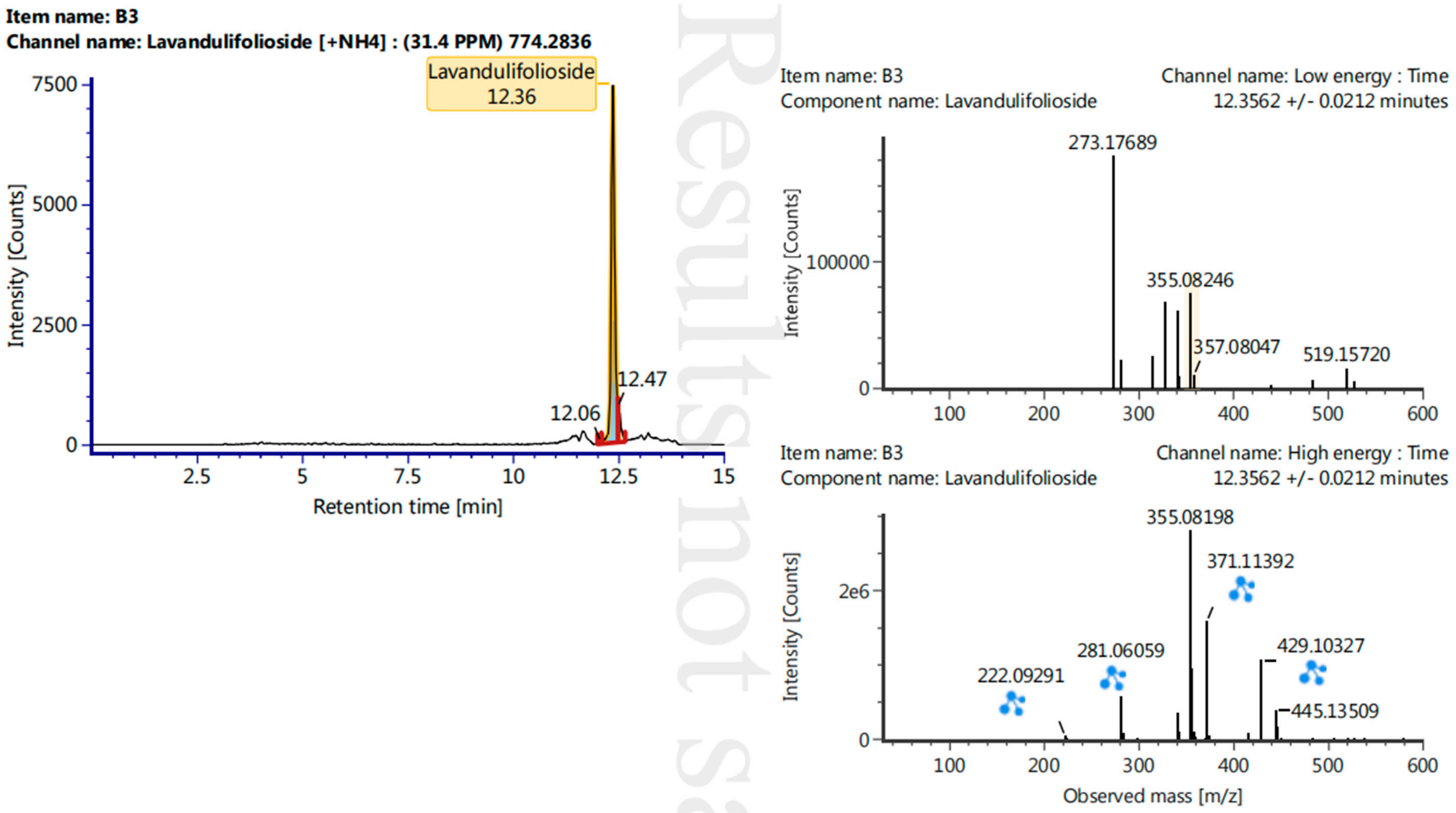

Results not Sa

Figure S46. MS/MS spectrum of Lavandulifolioside

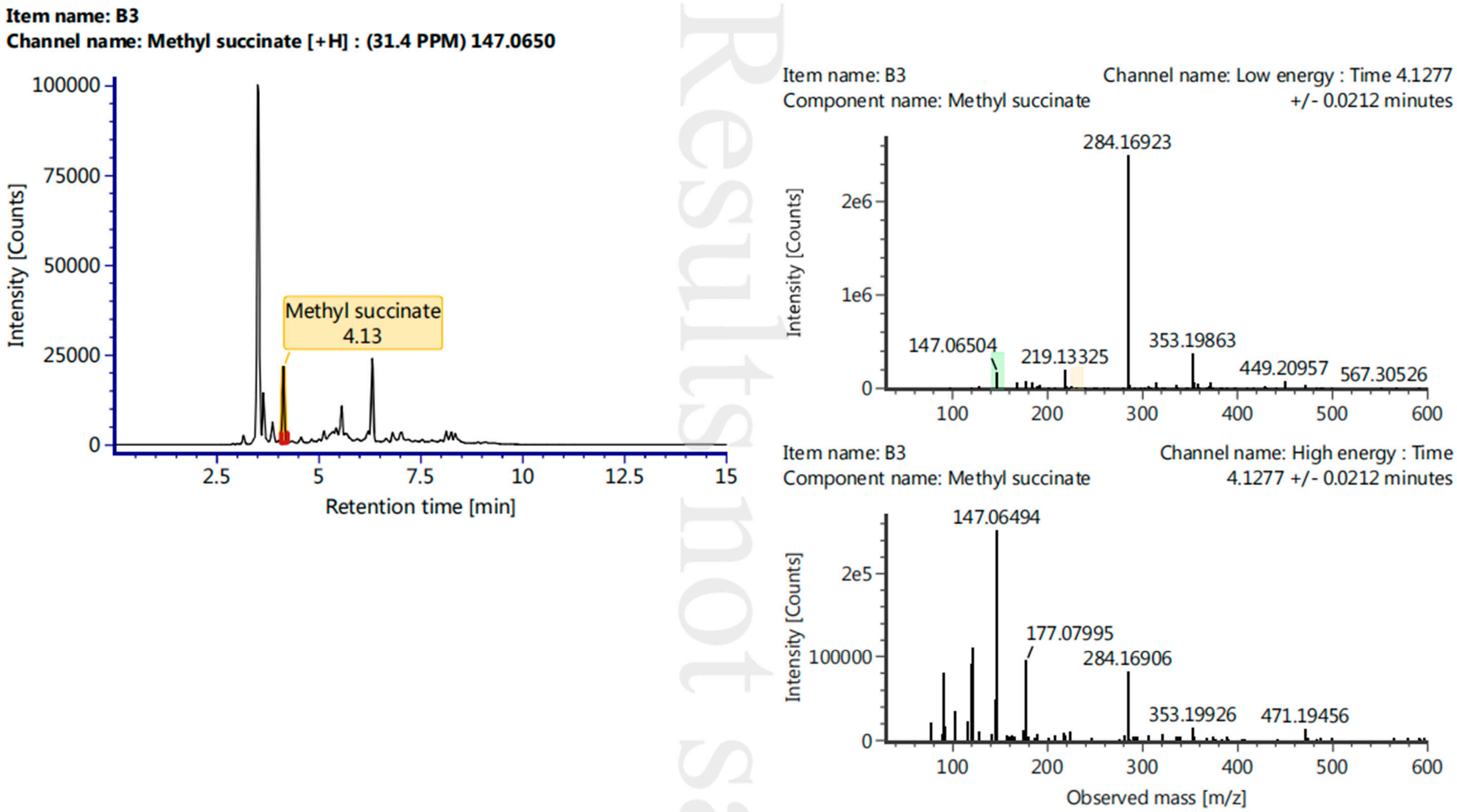

Figure S47. MS/MS spectrum of Methyl succinate

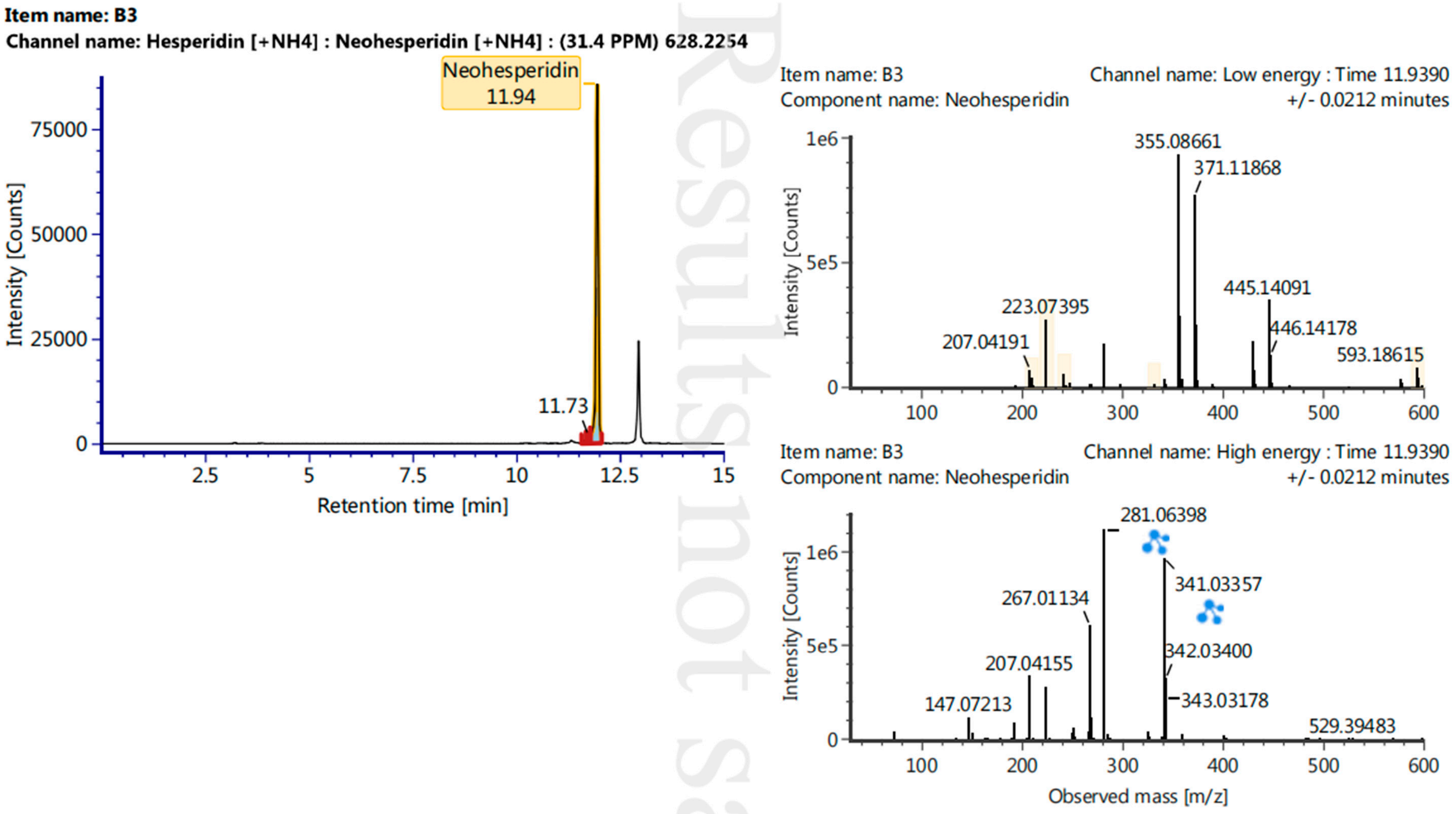

Results not Sa

Figure S48. MS/MS spectrum of Neohesperidin

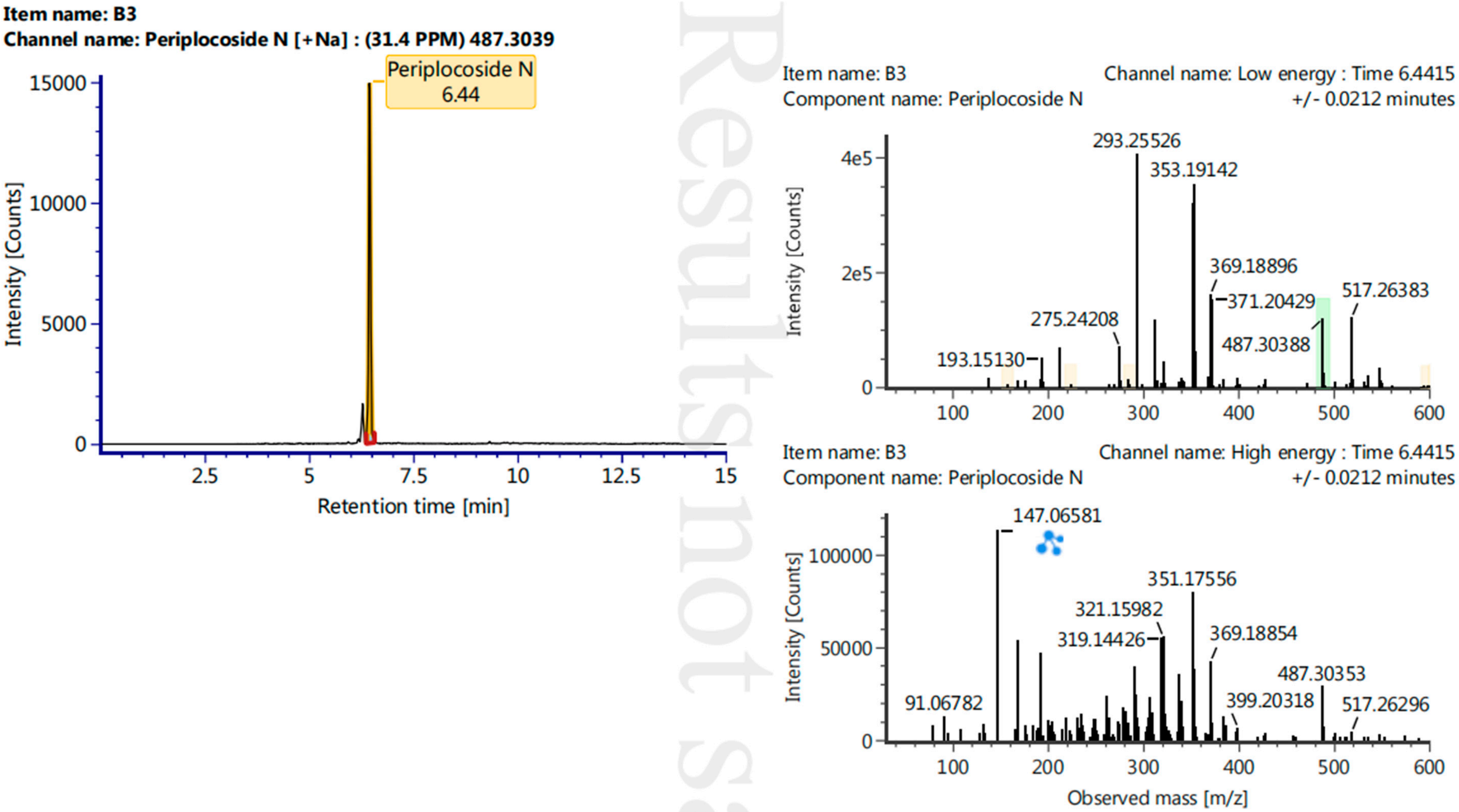

Figure S49. MS/MS spectrum of Periplocoside N

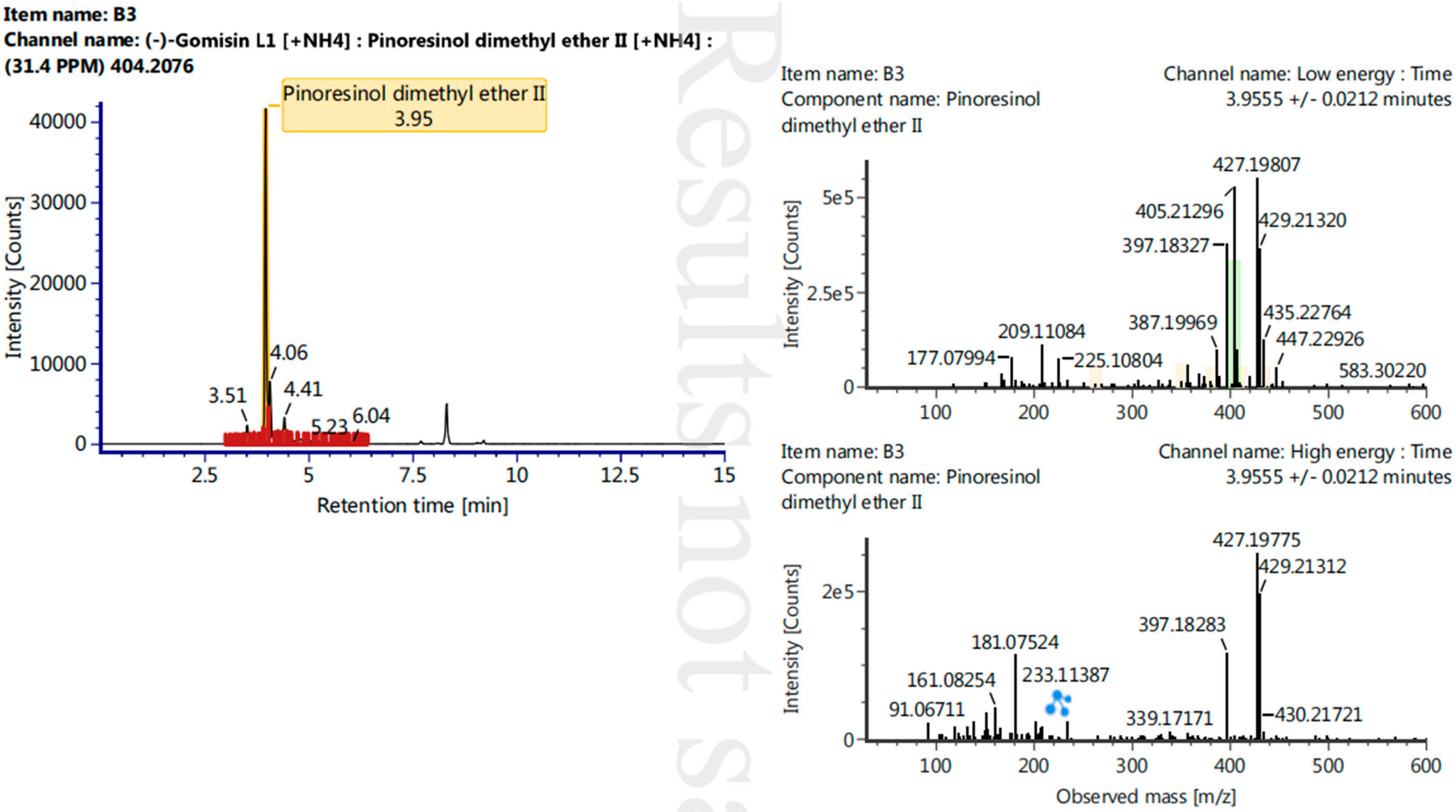

Figure S50. MS/MS spectrum of Pinoresinol dimethyl ether II

Results not Sa

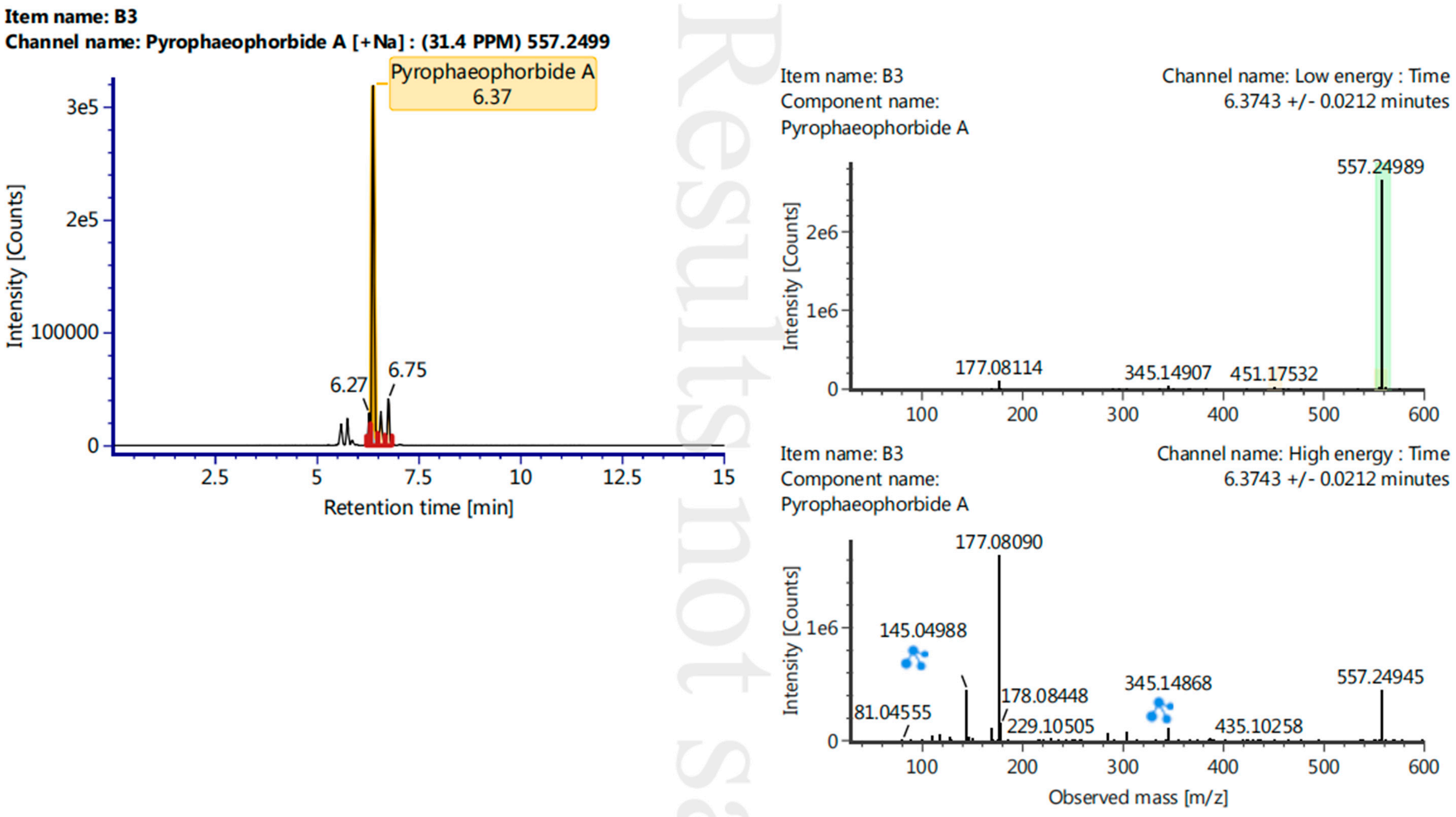

Figure S51. MS/MS spectrum of Pyrophaeophorbide A

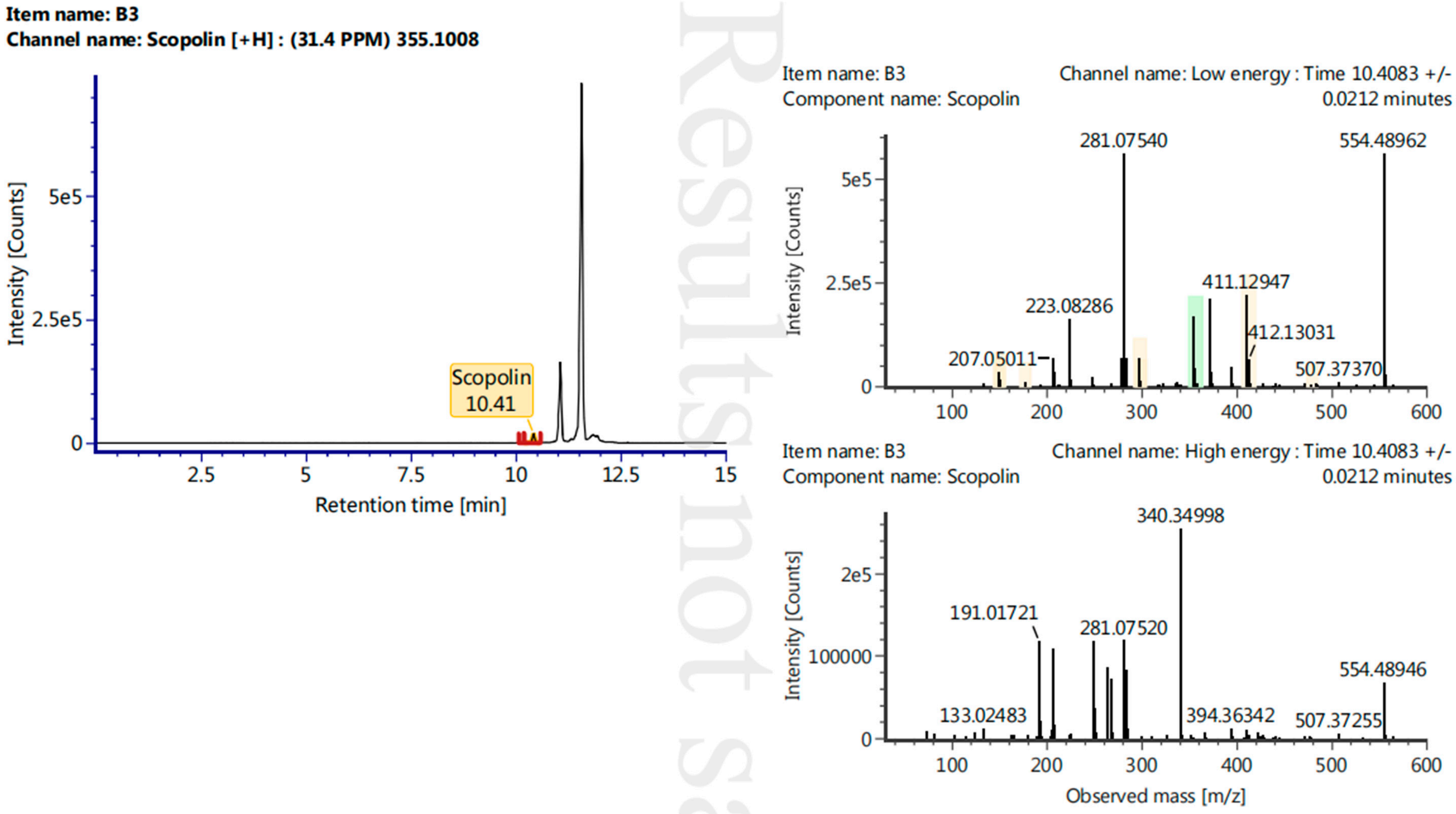

Results not Sa

Figure S52. MS/MS spectrum of Scopolin

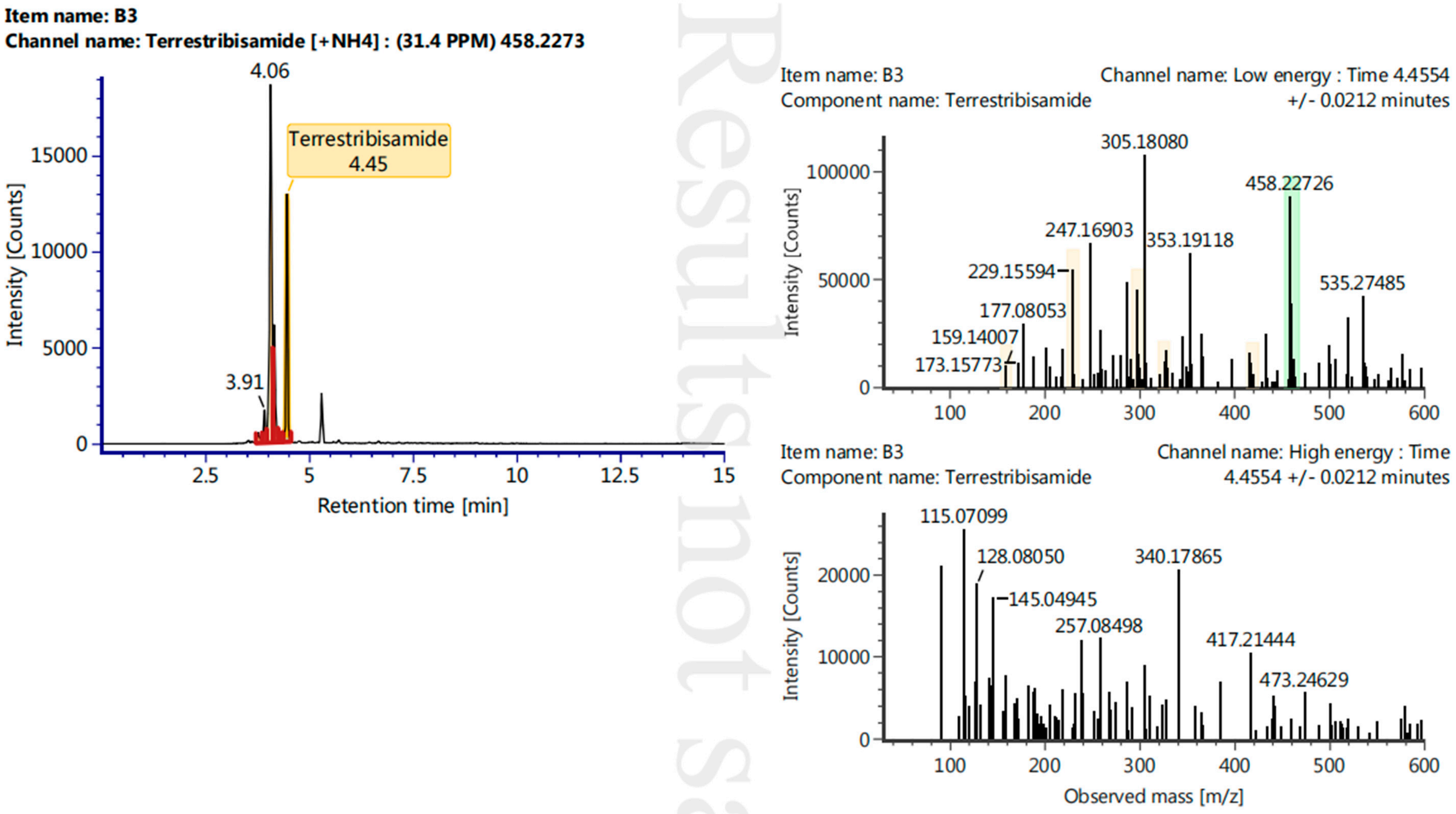

Figure S53. MS/MS spectrum of Terrestribisamide

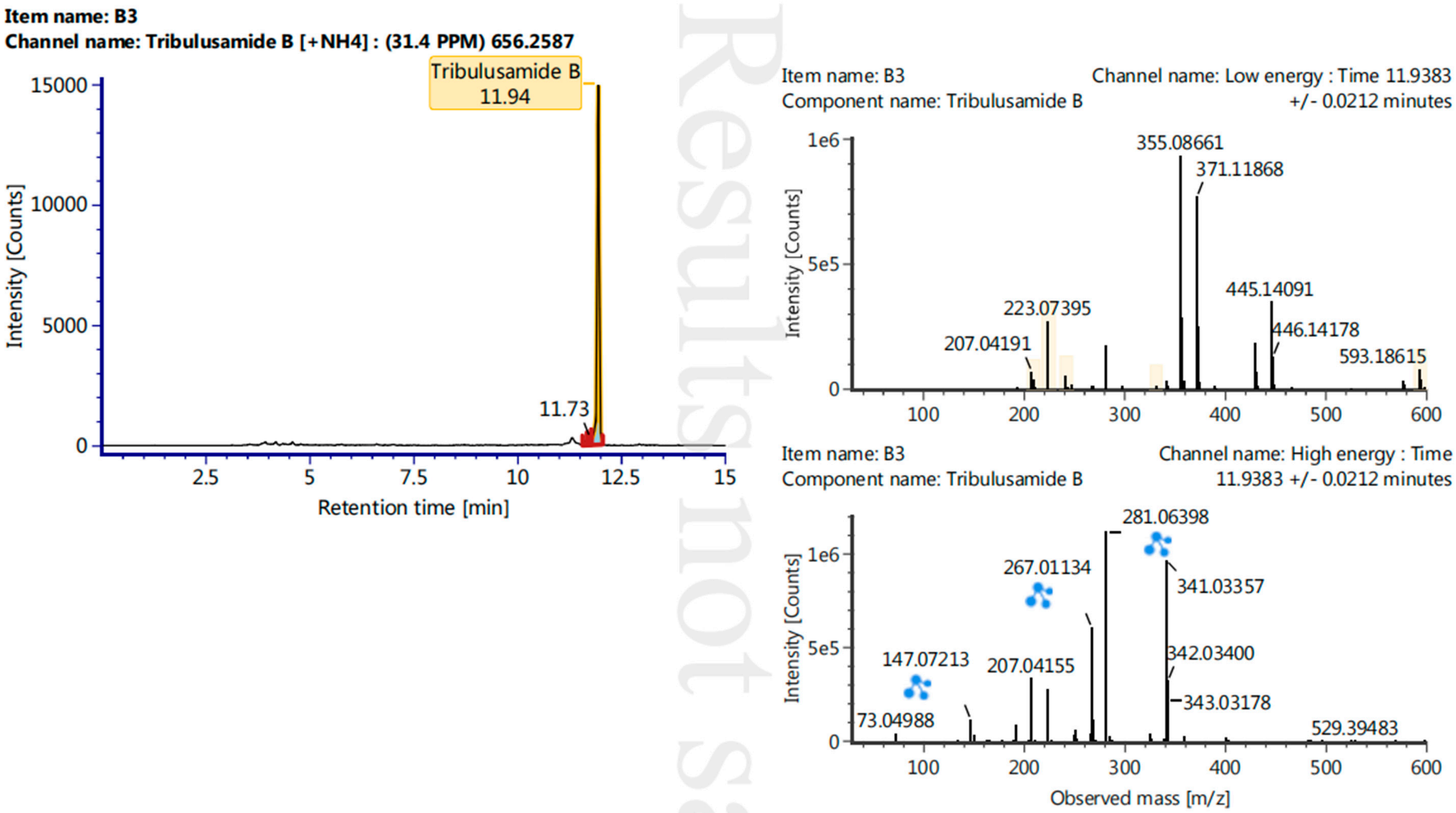

Figure S54. MS/MS spectrum of Tribulusamide B
